# Correlations of Behavioral Deficits with Brain Pathology Assessed through Longitudinal MRI and Histopathology in the *Hdh*Q150/Q150 Mouse Model of Huntington’s Disease

**DOI:** 10.1371/journal.pone.0168556

**Published:** 2017-01-18

**Authors:** Ivan Rattray, Edward J. Smith, William R. Crum, Thomas A. Walker, Richard Gale, Gillian P. Bates, Michel Modo

**Affiliations:** 1 King’s College London, Institute of Psychiatry, Department of Neuroscience, London, United Kingdom; 2 King’s College London School of Medicine, Department of Medical and Molecular Genetics, Guy’s Hospital, London, United Kingdom; 3 King’s College London, Department of Neuroimaging, Institute of Psychiatry London, United Kingdom; 4 University of Pittsburgh, Department of Radiology, McGowan Institute for Regenerative Medicine, Pittsburgh, PA, United States of America; Centre Hospitalier de l'Universite Laval, CANADA

## Abstract

A variety of mouse models have been developed that express mutant huntingtin (mHTT) leading to aggregates and inclusions that model the molecular pathology observed in Huntington’s disease. Here we show that although homozygous *Hdh*Q150 knock-in mice developed motor impairments (rotarod, locomotor activity, grip strength) by 36 weeks of age, cognitive dysfunction (swimming T maze, fear conditioning, odor discrimination, social interaction) was not evident by 94 weeks. Concomitant to behavioral assessments, T_2_-weighted MRI volume measurements indicated a slower striatal growth with a significant difference between wild type (WT) and *Hdh*Q150 mice being present even at 15 weeks. Indeed, MRI indicated significant volumetric changes prior to the emergence of the “clinical horizon” of motor impairments at 36 weeks of age. A striatal decrease of 27% was observed over 94 weeks with cortex (12%) and hippocampus (21%) also indicating significant atrophy. A hypothesis-free analysis using tensor-based morphometry highlighted further regions undergoing atrophy by contrasting brain growth and regional neurodegeneration. Histology revealed the widespread presence of mHTT aggregates and cellular inclusions. However, there was little evidence of correlations between these outcome measures, potentially indicating that other factors are important in the causal cascade linking the molecular pathology to the emergence of behavioral impairments. In conclusion, the *Hdh*Q150 mouse model replicates many aspects of the human condition, including an extended pre-manifest period prior to the emergence of motor impairments.

## Introduction

The cause of Huntington’s disease (HD) has been identified as the abnormal expansion of a CAG repeat in exon 1 of the huntingtin gene (*HTT*), that is transmitted in an autosomal dominant fashion [1]. This CAG expansion encodes a polyglutamine tract in the huntingtin protein (HTT), that is prone to aggregate and eventually manifests in neurodegeneration [2]. Nevertheless, the causal chain between the HD mutation and the development of disease signs remains poorly understood [3]. By its nature, HD is a variable disease and this, coupled with the scarcity of diseased tissue during the early stages, impede efforts to link brain atrophy and motor/behavioral dysfunction to a molecular pathology [4]. Pre-manifestation markers of the condition (i.e. biological changes that occur prior to symptom-based diagnosis) have been identified as subtle brain changes on magnetic resonance images (MRI) that are predictive of disease burden [5–8]. Rodent models of HD provide an excellent experimental system to thoroughly investigate these MRI-based biomarkers and associate these with cytoarchitectural and molecular changes in a controlled fashion [9].

Transgenic mouse models include the R6 [10] and N171Q82 [11] lines that express N-terminal fragments of HTT, as well as the YAC128 [12] and BACHD [13] lines that express a mutant version of the full-length protein as artificial chromosomes. The genetic basis of HD is more precisely recapitulated in the knock-in models, which possess an elongated CAG repeat that has been inserted into mouse *Htt*, or in which exon 1 of mouse *Htt* has been replaced with a mutant version of human exon 1 *HTT* with an expanded CAG repeat [14]. The *Hdh*Q150 knock-in model contains a highly expanded CAG repeat in the mouse *Htt* gene of a comparable length to that present in the R6/2 mice [15]. The model develops a more slowly progressing phenotype, which is advantageous for capturing early pre-manifest events [16]. It exhibits clear HD-like progressive behavioral phenotypes, HTT-positive nuclear and cytoplasmic inclusions and abnormal cytopathology [15–17]. Interestingly, the late-stage phenotypes of R6/2 mice at 12–14 weeks and *Hdh*Q150 mice at 90 weeks of age are highly comparable [18–24]. The R6/2 mice are a model for the incomplete splicing of the *HTT* gene that occurs in all full-length mouse models of HD resulting in a small exon 1 –intron 1 polyadenylated mRNA that encodes and exon 1 HTT protein [25].

Although there is an extensive description of the molecular pathology in the *Hdh*Q150 mice [18, 20, 26, 27], only a few studies have investigated the behavioral phenotypes [16, 26, 28, 29] or tissue changes by MRI [28]. To provide a link between molecular pathology with behavioral performance, as well as structural brain changes, a correlational analysis in the same cohort of animals is required, as we have previously described for the N-terminal fragment models R6/2 [30] and R6/1 [31]. To maintain face validity of the correlations between mouse models and the human condition, outcome measures in models should provide information that is relevant to the human disease [9, 32]. As HD affects the cognitive and motor abilities of patients, behavioral tasks encompassing motor, cognitive and emotional abilities should be evaluated longitudinally in the same animals to determine if these undergo progressive changes. To determine changes in brain structure over time, similar to human patients, MRI can be used to non-invasively visualize brain anatomy. Using sophisticated image analysis, it is possible to compare subtle sub-regional changes between experiment groups, but also over time [31, 33–35]. Based on these assessments, it is therefore possible to determine if subtle anatomical changes precede the clinical horizon at which clinical signs are diagnosed as HD [36].

Thorough characterizations of the pathological transition are essential for establishing a causal relationship between molecular pathology, tissue changes and resulting behavioral impairments. Here, we describe the longitudinal assessment (94 weeks / 2 years) and the emergence of behavioral dysfunctions on cognitive, motor and emotional tasks with concomitant measurements of brain atrophy by MRI, as well as post-mortem histopathological analyses. Correlational analyses of time course changes in these measurements were performed to indicate potential links between molecular pathology, tissue atrophy and behavioral performance, as well as an indication to pre-manifest MRI-based biomarkers.

## Methods

### Ethical statement

All procedures were performance in accordance with the ethical review procedures of King’s College London and carried out according to the Animals (Scientific Procedures) Act 1986 under the Home Office License (70/6445).

### Experimental design

**Fig 1:**
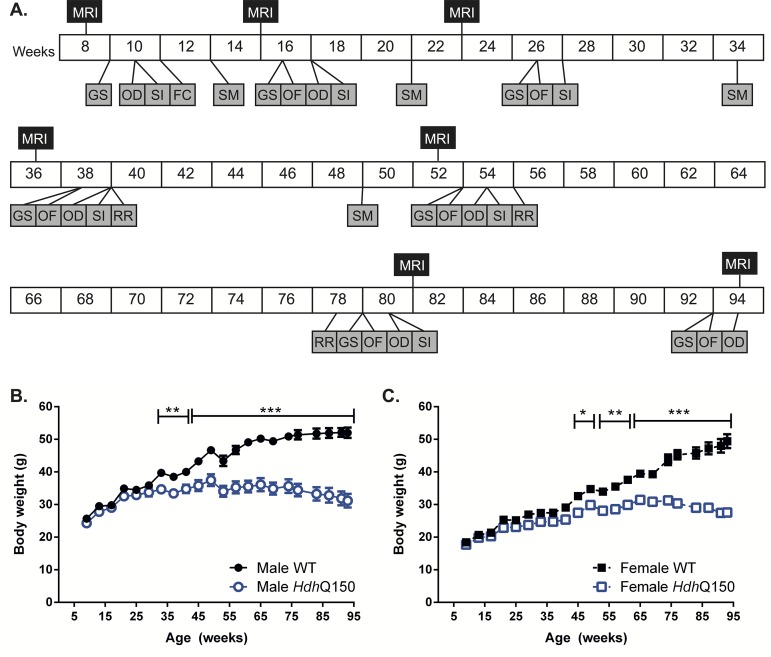
Experimental design and body weight measures. (A) Full experimental design from 8 to 94 weeks of age. *In vivo* MRI was taken, serially, at 8, 15, 23, 36, 52, 81 and 94 weeks. Behavior was assessed throughout the study at the weeks specified: GS = grip strength; OD = odor discrimination; SI = social interaction; FC = fear conditioning; SM = swimming T-maze; OF = behaviors in an open field; RR = rotarod. Body weight change in WT and *Hdh*Q150 male (B) and female (C) mice over time. All data presented as means ± SEM; *p < .05, **p < .01, ***p < .001.

Wild type (WT) and *Hdh*Q150 mice of both genders were included in a longitudinal study that repeatedly evaluated behavioral and tissue changes from 9 until 94 weeks of age when animals were perfusion fixed for post-mortem histopathological evaluations (Fig 1A). Motor-related behaviors consisted of grip strength, locomotor activity in an open-field, and rotarod testing. Non-motor-related behaviors entailed learning in a swimming T-maze; fear conditioning, odor discrimination, and social interaction. Concomitant to these evaluations, T_2_-weighted MR images were acquired to measure anatomical differences, with histological analyses determining neuronal loss and the presence of mHTT inclusions in the striatum and cortex at the end of the study. All procedures (behavioral, MRI and histological) were conducted on the same, single cohort of animals.

### Animals

Homozygous *Hdh*Q150 mice were obtained through crossing heterozygous *HdH*Q150 CBA/Ca and C57Bl/6J lines, as previously described in detail [18]. Mice were genotyped and the CAG repeat size was measured [21]. The CAG repeat lengths for both alleles were 161.9 (± 5.1) and 182.42 (± 9.81). CAG repeats did not significantly differ between male and female *Hdh*Q150 mice. Littermates were divided into four groups: male wild-type (WT, n = 8); female WT (n = 9); male *Hdh*Q150 (n = 9); female *Hdh*Q150 (n = 10). For exact subject numbers included at each stage of the study see S1 Table.

All mice were housed under standard animal laboratory conditions with room temperature maintained at 21°C (±1) and kept on 12h light:dark cycle automatically. Cages had standard environmental enrichment (bedding and play tube). Standard chow diet and tap water were available *ad libitum*. Mice were group-housed according to gender, but genotypes were mixed within the cages. Body weight was measured monthly, as was body temperature, measured through an infra-red temperature reader (ThermoScan Instant Thermometer, Braun), reproducibly positioned under the thorax.

### Behavioral tests

#### Rotarod

Rotarod assesses motor coordination and integration, commonly used to evaluate motor impairment in animal models of HD [37]. A previous study has shown that change in rotarod performance is a relatively late event in homozygous *Hdh*Q150 mice, detectable at 18 months of age only [18]. Thus, motor coordination was tested on an accelerating rotarod at 39, 55 and 78 weeks of age, using a previously described protocol [30, 31]. In brief, mice were individually placed on an accelerating, rotating beam (4–40 rpm) for a maximum of 300 sec. Latency to fall from the beam (sec) was recorded as a measure of motor performance. Mice were exposed to three trials per day for four days (day 1 behavior was considered habituation to the test and the data was subsequently discarded). The whole apparatus was thoroughly cleaned with 70% industrial methylated spirit (IMS) between trials.

#### Open field

To account for locomotor activity, general ambulation was probed in an open-field arena at 16, 26, 38, 53, 79 and 93 weeks of age. As previously described [30, 31], the test began 2 h into the mouse’s dark cycle, conducted under red-light conditions. Mice were habituated to the test room conditions for 2 h, then individually placed into plain, featureless white arenas (50 x 50 x 50 cm, Engineering & Design Plastics Ltd., Cambridge, UK) for 30 min. Behavior was videotaped via a camera positioned above the apparatus. Activity (distance moved, cm) was later tracked and analyzed using EthoVision 7XT software (Noldus, Netherlands). Arenas were thoroughly cleaned with 70% IMS between trials.

#### Grip strength

Fine motor behavior and strength in digits has been demonstrated to be associated with disease burden and potentially provides a pre-manifest sign of the clinical horizon [38]. Grip strength capacity was assessed at 9, 16, 26, 38, 53, 79 and 93 weeks of age. The protocol allowed for the independent measurement of the strength of the forelimbs alone, as well as fore- and hindlimbs taken simultaneously [31]. Mice were held by the base of the tail and gently swung to allow the mice to grip the wire-mesh grid attached to a grip strength monitor (Bioseb In Vivo Research Instruments). Once the mice gripped the grid, they were gently pulled away from the apparatus. Maximum tension (g) before the mice released the grid was recorded. For both grip strength recordings (forelimb only, and fore- and hind limbs together), mice were tested thrice. The average performance was taken for analysis. The apparatus was thoroughly cleaned with 70% IMS between trials.

#### Swimming T-maze

Visuospatial cognition involving matching-to-sample tasks is known to be impaired in patients with HD [39]. To probe cognitive deficits associated with cortical dysfunction, animals were exposed to a visuospatial matching-to-sample and reversal task. Due to the weight loss of animals at later time points, a food reward task was avoided to prevent food restriction. Instead, an escape-learning paradigm was used in a swimming T-maze [31]. Cue learning, and the reversal of cue learning was assessed in a swimming T-maze at 13, 21, 34 and 49 weeks, using an adapted protocol [40]. The maze consisted of a horizontal arm (122 cm length, 12 cm width, 35 cm depth made from black, non-transparent plastic) bisected in the center by a vertical arm (60 cm length, 12 cm width, 35 cm depth), filled with water made opaque through the addition of a whitening agent (Marvel dried skimmed milk, Premier Foods, UK). The maze was thoroughly cleaned every third day, and the water changed. A transparent plastic square escape platform was submerged 0.5 cm, invisible under the water surface. Water temperature was maintained at ~21°C throughout the duration of the procedure.

For “cue learning”, the escape platform was pseudo-randomly located (location order picked randomly out-of-a-hat), at either the left or right end of the vertical arm and was cued to the presence of an illuminated desktop lamp directly over the escape platform. Mice were individually placed in the starting position (at the base of the vertical arm, opposite where it bisects the vertical arm) and allowed to swim to the junction of the two arms. At this juncture, they made a decision to swim either toward the light (i.e. escape), or away from the light (i.e. no escape before being rescued after 10 sec). Mice were dried between trials. Mice were exposed to 12 trails per day until they reached criterion (10 correct choices out of 12 successive trials, 83.3% correct rate), whereby they began the second part of the trial, termed “cue reversal” learning. For cue reversal training, the desktop lamp cue was now situated at the opposite arm to the escape platform. Thus, mice were forced to re-learn the escape conditions. Cue reversal training was run until the mice reached criterion (again, 83.3% correct rate), at which point the assessment was complete.

#### Fear conditioning

Cued and contextual fear conditioning was used to probe cognitive function in these mice at 11 weeks of age, using an adapted protocol [31, 41]. Experiments were conducted using a standard fear conditioning system (TSE-Systems, Germany). The test was divided into three days, day 1 “training”, day 2 “cue recall and extinction” and day 3 “context recall”.

*Day 1 “training”*: The operant chamber was scented using a solution of 79.5% water, 19.5% ethanol, 1% vanilla extract. Mice were individually placed in the arenas and exposed to the conditioning protocol; three pairings of a conditioned stimulus (30 sec auditory tone), and an unconditioned stimulus foot-shock (0.5 mA, 1 sec duration).

*Day 2 “cue recall and extinction”*: The operant chamber was thoroughly cleaned with 50% ethanol to remove all vanilla extract odor. Monochrome patterned wall inserts and a plain white floor insert were added to change the context of the operant chamber. Mice were individually placed in the chamber and subjected to 25 exposures of the conditioned stimulus (auditory tone), but no foot shock, in order to measure cued recall and extinction of conditioned fear.

*Day 3 “context recall”*: The operant chamber was returned to the identical context as for Day 1 with mice being placed in the operant chamber for 5 min with no conditioned or unconditioned stimuli to determine contextual recall of conditioned fear.

Throughout the duration of these tests, behavior was videotaped by a camera positioned above the apparatus. Mouse immobility (a complete absence of movement except breathing) was considered a measure of fear, scored by an investigator blinded to the experimental groupings. An inter-rater reliability of >95% confidence was achieved [31]. For cued recall and extinction on Day 2, immobility over five summed conditioned stimulus (CS) exposures (giving a total of 5, 30 sec blocks) was used for analysis. For contextual recall on Day 3, immobility was expressed over the entire 5 min trial.

#### Odor discrimination

This test was conducted to probe olfactory function of mice at 10, 17, 39, 54, 80 and 94 weeks. Mice were habituated to plain white arenas (50 x 50 x 50 cm), for 10 min/day for two days prior to starting the test [31]. On the test day, two samples of mouse litter were available for investigation over a total of 5 min. One sample being clean litter, the other being soiled litter from unfamiliar sex-matched mice. Samples of litter were held in up-turned Eppendorf tubes with 0.5 cm of the tips removed to allow the animal to smell the contents. Behavior was videotaped from above and subsequently analyzed. Time spend investigating the tubes (nose pokes directly at the aperture of the tubes) was quantified by an investigator blinded to both the samples and experimental groupings. Preference for the soiled litter was expressed as percentage of time investigating soiled sample out of total time investigating either sample. An inter-rater reliability of >95% was achieved. Arenas were thoroughly cleaned with 70% IMS between trials.

#### Social interaction

Social behaviors, as well as the ability to discriminate novelty, was tested at 10 weeks of age using an adapted protocol [31, 42]. Test mice were habituated to plain white arenas (50 x 50 x 50 cm) for 1 h. Two corrals (up-turned pen holders, Staples, UK) were then positioned inside the arenas in reproducible positions, and the test mice were allowed to habituate to the corrals for a further 30 min. During “exposure 1”, a sex-matched, 8 weeks old C57Bl/6 mouse was positioned in one corral and the test mouse was allowed to investigate, but not physically interact with the mouse for 5 min. This now “familiar” mouse was removed, and following an inter-trial-interim of 30 min “exposure 2” was conducted, whereby the “familiar” mouse was replaced back into the same corral and a different, “novel” C57Bl/6 mouse was placed in the other corral. The test mouse was free to investigate either the familiar or novel mouse. Behavior was videotaped from above and subsequently analyzed. Time spent investigating the familiar and/or novel mice was measured by an investigator blinded to the experimental groups. An inter-rater reliability of >95% was achieved [31]. Arenas and corrals were thoroughly cleaned with 70% IMS between trials.

### Magnetic resonance imaging

#### In vivo longitudinal MRI

WT and *Hdh*Q150 mice were scanned *in vivo*, serially, a total of seven times at 8, 15, 23, 36, 52, 81 and 94 weeks of age. Mice were anaesthetized using 5% isoflurane along with a combination of medical air (0.7 L/min) and oxygen (0.3 L/min). Once fully anesthetized, mice were positioned and fixed into a plastic frame, where anesthetic was administered through a facemask. Mice were maintained under anesthesia, typically between 1–2% isoflurane for the duration of the scanning. Temperature was maintained through a homeostatic heating airflow system and breathing rate monitored through a respiration balloon positioned under the thorax (Small Animal Instruments, New York, USA). Post-scanning, but prior to recovery, mice were administered 0.1 mL saline i.p. to abate dehydration.

Images were acquired on a 7 T horizontal bore MRI system (Varian, Paolo Alto, California, USA), with a 100 Gauss gradient set insert and a 39 mm bore (transmission and receiver) radiofrequency coil (Rapid, Germany). The scanner was controlled through VnmrJ software (Varian, Paolo Alto, California, USA). Correct positioning of the mouse within the RF coil was confirmed through a series of scouting images. A Multi-Echo-Multi-Slice (MEMS) scan was then acquired (*TR* = 2500 msec, *TE* = 10 msec, echo train = 8, averages = 4, matrix = 128 x 128, FOV = 20 x 20 mm, 156 μm in plane resolution, 30 coronal slices at 0.5 mm thickness, 21 min acquisition time). The typical signal-to-noise ratio (SNR) = 5.4, the typical white:grey matter ratio (WGR) = 1.25. Coronal slices were positioned based on a reproducible anatomical marker (the most visibly posterior part of the cerebellum).

Post-acquisition, all eight echoes were summed into a single structural image set. As previously described [30, 31], these images were used to manually delineate neuroanatomical structures in JIM Ver. 5.0 (Xinapse Systems, Alwincle, UK). Regions-of-interest (ROIs) consisted of whole brain, cortex, striatum, hippocampus, and corpus callosum. ROIs were delineated by two investigators blinded to the experimental groupings, and intra- and inter-rater reliability was consistently ≥ 95% confidence. Details of neuroanatomical inclusion criteria and delineation guidelines were identical to those described previously [30]. All information outside of the ROIs was subsequently masked out, the ROIs were then individually saved in NIFTI format. Volumetric data were calculated and processed semi-automatically using Python Ver.2.6 (Python Software Foundation). To measure changes in T2 relaxivity (reflective of tissue composition), maps of T2 signal intensity were obtained through a mono-exponential fit of the eight echoes. The ROIs were superimposed onto the maps of T2 signal intensity allowing for the generation of mean T2 relaxation times within each ROI. A small circular ROI was taken for cheek muscle tissue T2 relaxivity in order to act as an internal control measure.

#### Ex vivo MRI

Following the final *in vivo* MRI scan, at 94 weeks of age, mice were anaesthetized using a terminal anesthesia Euthatal (Marial, Harlow, UK), and then transcardially perfused with heparinized saline (50 units/ml), followed by Parafix (4% paraformaldehyde, Pioneer Research Chemical Ltd., Essex, UK). Whole heads were removed and submerged in Parafix and kept at 4°C until *ex vivo* imaging. Post-mortem *ex vivo* MRI scans were taken. These images were higher resolution compared to the *in vivo* scans, and did not suffer the potential artifacts which can arise from live scanning (e.g. physiological movement), therefore allowing a more precise measurement of more subtle changes in brain structures. The scanning set-up was identical to that used for *in vivo* imaging. Correct positioning of the mouse head within the RF coil was confirmed through a series of scouting images. An MEMS sequence was then acquired (*TR* = 3000 msec, TE = 10 msec, echo train = 8, averages = 22, matrix = 192 x 192, FOV = 19.2 x 19.2 mm, 100 μm in plane resolution, 35 coronal slices at 0.5 mm thickness, acquisition time for scan was ~3.5 hours). Typical SNR = 11.96, typical WGR = 1.54.

#### Tensor based morphometry

An un-biased whole-brain comparison of WT and *Hdh*Q150 mice at each imaging time-point was performed using an automated image processing pipeline [43]. All scans were first registered with 6 degrees of freedom (dof) (to remove differences in position and orientation using a rigid-body assumption) and 9 degrees of freedom (to optionally remove global differences in scale using a growth model based on uniform scaling), using a population-based approach which has proven robust in rodent imaging applications [33]. Then each scan was non-rigidly registered to the WT mean at the same time-point using a high-dimensional fluid registration technique [44–46]. This technique models the coordinate mapping between scans as the flow of a viscous fluid and can successfully map large structural displacements, as well as smaller localized changes in shape. This technique has previously been applied in other rodent models, e.g. structural remodeling in stroke [43]. The fluid registration results in a dense displacement field, which maps each point in the original scan to the corresponding point on the reference mean. From this map, an estimate of apparent volume difference (the Jacobian determinant) between the scan and the WT mean at each voxel can be obtained. TBM analysis then applies voxel-wise non-parametric t-tests to these volume difference estimates to determine the location of statistically significant differences in brain tissue volume of *Hdh*Q150 compared with WT. Significance levels are corrected for multiple comparisons across voxels using the False Discovery Rate (FDR). The analysis following 6 dof registration finds absolute volume differences (i.e. the fluid registration includes differences in brain size and the analysis finds absolute volume difference between regions). Following 9 dof registration, volume differences are relative to whole brain volume, V (i.e. the fluid registration does not include differences in global brain volume and the analysis finds volume differences relative to whole brain volume). Collectively, these analyses allow for the comparison of WT versus *Hdh*Q150 at each time point (seven image sets *in vivo* and one *ex vivo*), as well as the age-related change within each genotype (comparing all other time points to the scan at 36 weeks of age when WT brains stopped growing). The image analysis approach is described in more detail in [43].

### Histology

Upon completion of *ex vivo* imaging, mice were perfusion-fixed with heparinized saline, followed by 4% paraformaldehyde (PFA). Brains were removed from the skulls, rinsed in phosphate buffered saline (PBS) and stored in 30% sucrose in PBS (+0.05% sodium azide) until sectioning. Coronal sections were taken serially at 50 μm thickness on a freezing microtome (HM430 Microm, Thermo Scientific) and stored at -20°C in tissue cryoprotective solution (30% Ethylene Glycol, 25% Glycerol and 0.5% Sodium Azide in PBS) until staining. Histological processing and data collection was performed identically to that described previously in [30].

#### Immunohistochemistry

Sections were washed in PBS prior to incubation for 30 min in 3% H_2_O_2_ in PBS to quench endogenous peroxidase activity. Non-specific binding was blocked with a 1 h incubation in 10% normal serum with 0.3% Triton X-100 in PBS. Sections were then incubated overnight at 4°C in primary antibodies against NeuN (1:500, Millipore, Watford, UK) or S830 (1:2000), raised against exon 1 HTT with 53 glutamines [47], prior to incubation in appropriate biotinylated secondary antibody (Vector, Peterborough, UK) for 2 h at RT, followed by 1 h incubation in an avidin-biotinylated-peroxide complex (1:100, Vector, Northampton, UK). 3, 3'-diaminobenzidine (Sigma-Aldrich, Poole, UK) was used as the chromagen.

#### Cortical thickness

Assessment of regional cortical atrophy was determined by thickness measurements of primary motor cortex (M1) and primary sensory cortex (S1) on NeuN-stained sections [30, 31]. In each region, 10 vertical lines were drawn covering all layers from the most dorsal horn of the corpus callosum to the pial surface. From these measurements, the mean length was calculated from 3 consecutive sections (approximately Bregma +1.10 mm).

#### Stereology of NeuN-stained sections

Unbiased stereological estimates of volume and neuronal number were obtained using StereoInvestigator software (Microbrightfield, Willston, VT). All stereological measurements were performed with the observer being blinded to the experimental grouping. The Cavalieri method was used to obtain unbiased estimates of striatal and M1 cortical reference volumes [48]. ROIs were defined by x1.6 magnification lens through reference to neuroanatomical landmarks. For both the striatum and M1 cortex, equally spaced sections (50 μm thickness each, 450 μm gap) were analyzed. As defined by Sadikot and Sasseville [49], sections contained within the striatum were sampled anteriorly from the first appearance of the genu of the corpus callosum (Bregma = +1.1 mm) to posteriorly at the first evidence of a hippocampal formation (Bregma = -0.94 mm). The dorsal and lateral boundaries consisted of the corpus callosum with the medial boundary being the lateral ventricles/internal capsule. For sections rostral to where the dorsal 3^rd^ ventricle has joined the lateral ventricles, ventral boundaries become lateral ventricles/globus pallidus. The striatal volume was sampled by 4–5 sections for both WT and *Hdh*Q150. M1 cortex was measured anteriorly from +1.1 mm bregma to posteriorly -0.94 mm bregma from layers II to VI, as defined in a stereotaxic atlas [50]. The absence of cortical layer IV (indicative of the S1 cortex) defined the lateral boundaries of M1, whereas medial boundaries consisted of the most dorsal part of the corpus callosum. M1 was sampled by 4–5 sections for both WT and *Hdh*Q150.

To obtain unbiased estimates of neuronal numbers, the optical fractionator was employed as a stereological probe (coefficient of error <0.1). Section thickness and neuronal counts were performed under oil immersion with the x100 objective (Zeiss) with a numerical aperture of 1.4. A sampling grid was applied appropriate to the structure measured (cortex = 200 μm x 200 μm, striatum = 400 μm x 400 μm) with a counting frame of 65 μm x 35 μm with a mean thickness of 18 μm. Guard zones of 0.5 μm were applied at the top and the bottom of each frame with a mean dissector height of 17 μm.

#### Quantitative analysis of S830

Evaluation of mHTT immunoreactivity in different brain regions was performed using an intensity-based measurement of S830 staining [30, 31]. Non-overlapping images (using fixed exposure and light intensities at x40) were obtained from 3 consecutive sections expressing the striatum or hippocampus, and 6 sections for the cortex. In total, 30 striatal (10 per section), 60 cortical (10 per section) and 36 hippocampal subregion (12 per section) images were taken. All images were captured in RGB using a live video camera (JVC, 3CCD, KY-F55B), mounted onto a Zeiss Axioplan microscope.

Staining intensity was quantified using threshold-based analysis software (Image Pro Plus, Media Cybernetics, IL, USA) assessing optical density of the immunoreactive product. Threshold levels were chosen based on the minimum level of transmitted light needed to detect the immunoreactive product on a scale of 0 (100% transmitted light) and 255 (0% transmitted light) for each pixel. Two levels were taken to measure dense, nuclear mHTT inclusions (90), and total aggregated mHTT staining (nuclear and extra-nuclear, 130); mean percentage immunoreactivity area per field of view (FOV) was recorded.

### Statistical analyses

Data was graphed using Prism Ver.5.0b (GraphPad Software, California, USA). Statistical analyses were calculated using SPSS Statistics Ver.20 (IBM, Portsmouth, UK). All data were screened for statistical outliers using Grubbs’ Test (GraphPad Software, California, USA). Grubbs’ test calculates a z ratio for each value within a given dataset (determined through subtracting each value from the group mean, and dividing it by the standard deviation). If the z ratio for any given value is greater than those proposed by Grubbs for that population size, it is considered an outlier. If this was the case, the value was excluded from analysis. Due to either animal loss, or occasional missing data samples and removal of statistical outliers (total absent data samples for male WT = 8, male *Hdh*Q150 = 9, female WT = 10, female *Hdh*Q150 = 9), the number of animals varied for each test at different time points. A table summarizing the number of subjects included for each measure and the reason for exclusion can be found in S1 Table.

The acquired datasets can be separated into those that were collected longitudinally, those acquired at a single time point, correlational analyses, as well as tensor based morphometry statistics.

#### Longitudinal datasets

These datasets refer to tests where data was collected at more than one time point and include: body weight assessment, rotarod, locomotor activity, grip strength measurements, swimming T-maze, odor discrimination, and social interaction, as well as measurements of volumetry and T2 relaxivity through MRI. It was not possible to compute a repeated measures ANOVA due to missing values at different measurement times. To probe the influence and interaction of genotype and gender across time, a three-way ANOVA was applied (Genotype, Gender and Age, as between-subject factors).

#### Repeated measures dataset

To determine whether mice exhibited cued fear conditioning and extinction (i.e. a form of re-learning that the CS is no longer aversive with repeated exposure), a repeated measures ANOVA was used (repeated Tone (CS) exposure as within-subject factor, Genotype and Gender as between-subject factors). Post-hoc tests with a Bonferroni correction for multiple comparisons were applied where appropriate. All main effects from ANOVAs can be found in S2 Table.

#### Datasets taken at a single time-point

These data refer to tests that were only conducted once during the study, such as histological measures taken through stereology. To probe the influence of genotype and gender on these measures, a two-way ANOVA was applied (Genotype and Gender as between-subject factors). Post-hoc tests with a Bonferroni correction for multiple comparisons were applied where appropriate. All main effects from ANOVAs can be found in S2 Table.

For the histological quantification of mHTT, there was an absence of immunoreactivity in the WT mice. This marker, within this group, was therefore not included in the analysis. To compare levels of mHTT across various brain regions, and probe the influence of gender on this measure a two-way ANOVA was applied (Region and Gender as between-subject factors). A post-hoc test was conducted for multiple comparisons, with a Bonferroni correction, comparing male and female *Hdh*Q150 mice at each brain region.

#### Correlative analyses

The Pearson Correlation Coefficient was used for all correlative analyses. To account for multiple correlations of the same data, the significance level was corrected using a Bonferroni correction by dividing the standard p value (0.05) by the number of comparisons made, resulting in an appropriate adjusted p value for each dataset.

#### Tensor based morphometry statistics

As described in [43], a non-parametric two-tailed *t*-statistic, assuming unequal variance between groups, is computed at each voxel (approximately 42,000) in the brain. Permutation-testing is used to assess significance. The effective number of permutations at each voxel is increased by pooling null-distribution statistics from other voxels to allow for accurate multiple comparisons correction using the False Discovery Rate (FDR) [51]. The minimum number of required permutations is approximately (number-of-voxels / FDR-significance-level) = 42,000 / 0.05 = 840,000.

## Results

### Lack of weight gain in *Hdh*Q150 mice

A decrease in animals’ weight is a general physiological indicator of poor health. There was a steady weight gain in WT and *Hdh*Q150 animals for males (Fig 1B) and females (Fig 1C). Male *Hdh*Q150 mice exhibited normal weight gain until week 35 [F(Genotype X Age)_21;788_ = 31.704, p<0.001), and female *Hdh*Q150 mice until 45 weeks of age [F(Genotype X Gender)_1;788_ = 15,772, p<0.001] (S2 Table for all statistical results). Although there was no significant weight loss in both *Hdh*Q150 genders, there was no longer a weight gain, as observed in WT of both genders [F(Genotype)_1;784_ = 1197.939, p<0.001]. Body temperature decreased with age [F(Age)_20;754_ = 113.1, p < .001], with no genotype effects for male or females [F(Genotype X Gender) _1;754_ = 0.022, n.s.]. However, females’ body temperature was overall higher than males by ~1°C [F(Gender) _1;754_ = 240.471, p<0.001].

### A slowly progressive motor function decline

**Fig 2:**
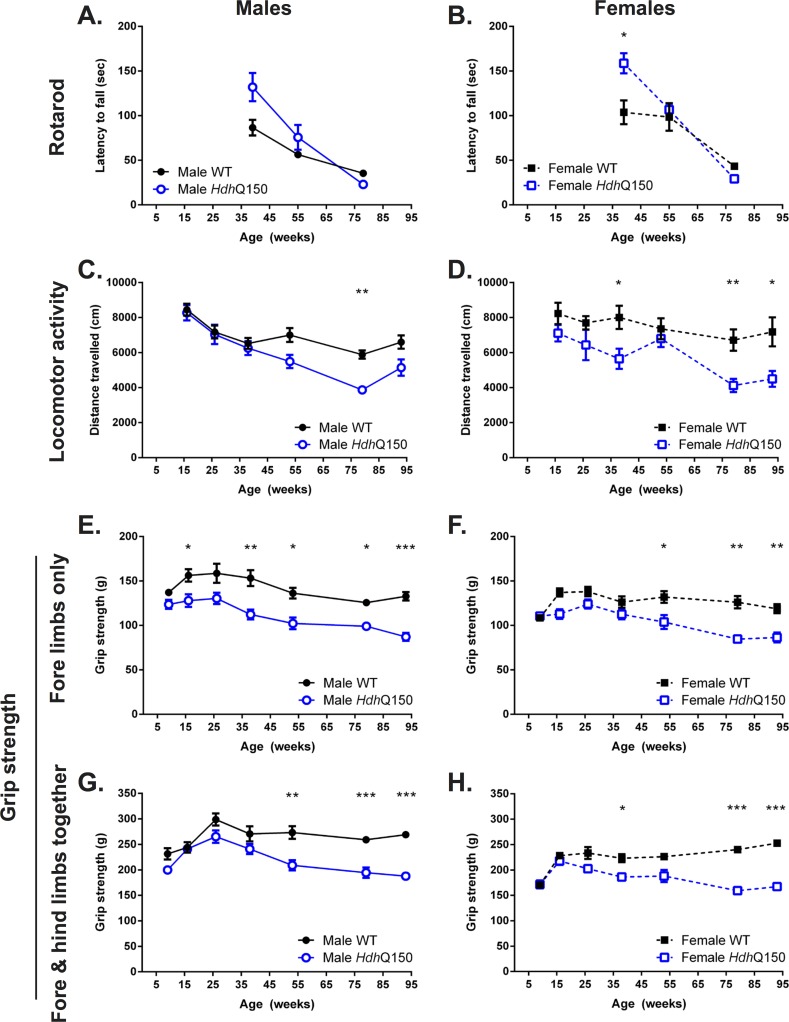
Performance at motor-related tasks. Both male (A) and female (B) *Hdh*Q150 mice performed better on the rotarod compared to controls, but performance of all animals deteriorate with no difference between *Hdh*Q150 and controls beyond 55 weeks of age. *Hdh*Q150 male (C) and females (D) developed a general age-related hypoactivity in an open-field arena. Fore-limb grip strength capactiy was lower in *Hdh*Q150 male mice from as early as 16 weeks of age (E), whereas a deficit in female *Hdh*Q150 was detectable from 53 weeks (F). Similarly, fore- and hind-limb grip strength, taken together, was progressively lower in both male and female *Hdh*Q150s versus WTs (G & H). All data presented as means ± SEM; *p < .05, **p < .01, ***p < .001.

One of the hallmarks of the clinical horizon of Huntington’s disease is the onset of motor dysfunction. The rotarod is a commonly used task to assess motor coordination. Interestingly, here at 39 weeks of age, *Hdh*Q150 animals of both males (Fig 2A) and females (Fig 2B) performed better on this task than WT [F(Genotype) _1;106_ = 8.211, p<0.01], with a gradual decline of performance by 55 weeks of age [F(Genotype X Age) _2;106_ = 9.793, p<0.001]. At the late stage WT and *Hdh*Q150 did not exhibit a significant difference in performance, although their latency to fall from the rod further decreased [F(Age) _1;106_ = 74.303, p<0.001].

General ambulant locomotor activity (open field) diverged with the rotarod performance. Notably, for male subjects there was no significant difference in performance up to 38 weeks of age, whereas for females, there was already evidence of a decreased performance that accentuated with time [F(Genotype X Gender) _1;213_ = 3.918, p<0.05] at the start of testing (16 weeks of age). At the final time point (93 weeks of age), male *Hdh*Q150 revealed a 34% decreased in locomotor activity (Fig 2C), whereas female *Hdh*Q150 animals’ performance showed a 39% attenuation compared to controls (Fig 2D). Genotype was a major factor in locomotor activity [F(Genotype) _1;213_ = 40.264, p<0.001], as was age [F(Age) _1;213_ = 14.481, p<0.001].

For grip strength, which measures fine motor behavior and muscle strength, male *Hdh*Q150 mice already revealed a significant difference to WT strength at 16 weeks of age (the earliest time point tested). This deficit persisted, but only started to decline after 26 weeks of age (Fig 2E). Although female *Hdh*Q150 developed also a deficit in grip strength (Fig 2F), the emergence of this was delayed and only became apparent after 38 weeks of age with a gradual, but steady decline. The deficit in male *Hdh*Q150 was 7% greater at the final time point compared to females. The combined fore and hind limb strength were comparable to WT up until 38 weeks of age, when a gradual decrease was observed for both male (Fig 2G) and female *Hdh*Q150 mice (Fig 2H). However, it is important to note that this decrease brought *Hdh*Q150 animals back to the level of strength they exhibited at 16 weeks, indicating that the magnitude of decrease is different in nature compared to that observed for locomotor activity, where performance was 55% less than at 16 weeks of age.

### Lack of a robust cognitive impairment

**Fig 3:**
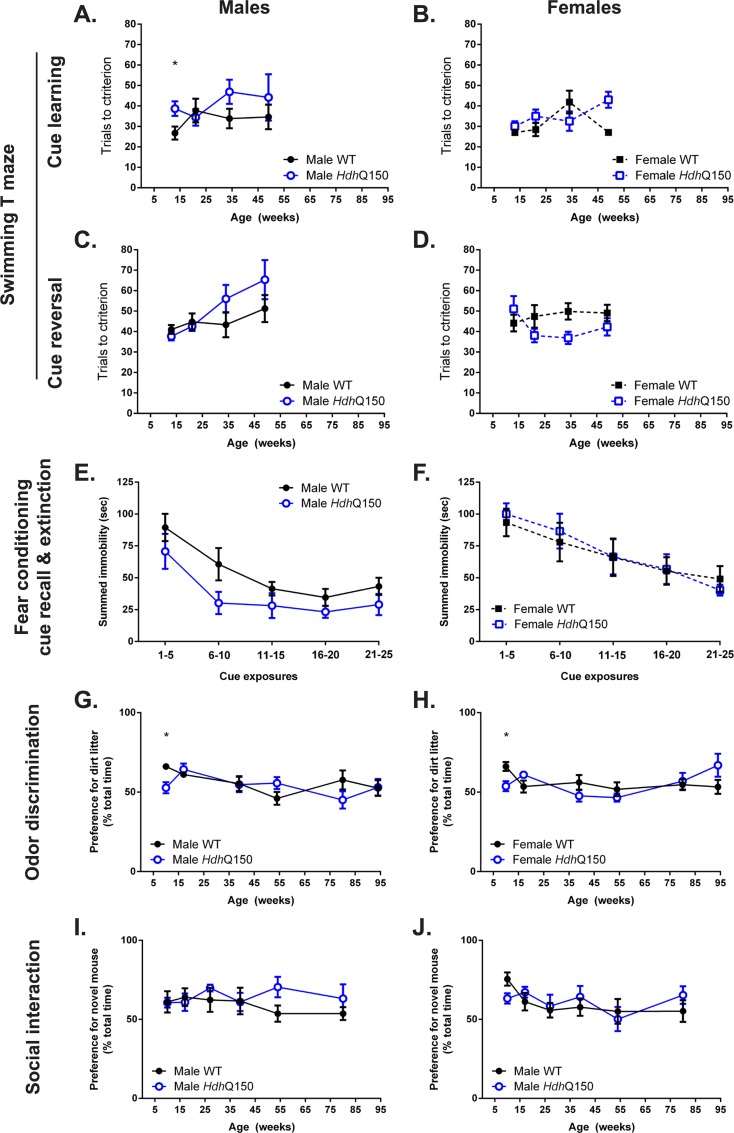
Performance at non-motor-related tasks. Cued learning (A & B) and cue reversal learning (C & D), assessed in a swimming T-maze, was generally intact in *Hdh*Q150 mice versus WTs with the exception of an apparent deficit in cued learning in male *Hdh*Q150 mice at 13 weeks only (A). Cue recall and extinction was similar between WT and *Hdh*Q150 mice irrespective of gender at 11 weeks of age (E & F). Both male and female *Hdh*Q150 had a lower preference for soiled little, versus WTs, at the odor discrimination task at 10 weeks only (G & H). There was no difference in social interaction behaiovurs at any age investigated (I & J). All data presented as means ± SEM; *p < .05.

Cognitive deficits are also associated with the clinical symptomatology of HD, but little is known about the presence, as well as the evolution, of cognitive performance in mouse models of the disease. A matching-to-sample task was implemented in the swimming T-maze to probe visuospatial cognition. At 13 weeks of age, male *Hdh*Q150 mice took significantly longer to learn the task (Fig 3A), whereas females did not exhibit a deficit (Fig 3B). Male *Hdh*Q150 mice consistently performed worse, but this difference was not significant. Females in contrast did not show a deficit in this task until 34 weeks, but performed significantly worse than WT at 49 weeks of age. Cue reversal did not initially show any impairment, but by 34 weeks of age male *Hdh*Q150 mice performed poorer compared to WT (Fig 3C), although the high variability in *Hdh*Q150 did not reveal a statistically significant result. Female *Hdh*Q150 actually showed a significantly better performance at 34 weeks of age, although performance was again comparable to WT at 49 weeks (Fig 3D). Males therefore demonstrated a mild deficit on this task, whereas females performed well. Acquisition of the swimming water maze was halted at 49 weeks of age as we previously observed a significant number of unexpected deaths following swimming maze performance in aged animals.

Additional cognitive tasks also failed to reveal a major deficit. Fear condition in male *Hdh*Q150 mice showed a slightly poorer performance compared to WT (Fig 3E), but the performance of female *Hdh*Q150 animals was equivalent to WT (Fig 3F). Odor discrimination, which is often considered an early biomarker in neurodegenerative disease [52, 53], revealed an early difference between WT and HdhQ150 at 10 weeks of age [F(Genotype X Age) _5;212_ = 3.028, p<0.05], but did not show any further performance differences in male (Fig 3G) or female *Hdh*Q150 mice (Fig 3H). Probing of social interaction failed to reveal any significant effects for male (Fig 3I) or female *Hdh*Q150 mice (Fig 3J), but there was an indication of a poorer performance for male mice beyond 39 weeks of age. Overall, there was a lack of robust evidence to indicate that *Hdh*Q150 mice exhibit impairments in these cognitive domains (i.e. visuospatial processing, memory, odor discrimination, social interaction).

### Performance on motor and cognitive tasks was mostly uncorrelated

The battery of behavioral assessments was designed to evaluate different domains and in normal animals should be orthogonal (i.e. independent of each other). This indeed was the case for the early time points (9–13 weeks of age; S3 Table). Between 16–54 weeks, the only correlation that survived Bonferroni correction was for animals where grip strength in the forepaws correlated with grip strength of all four limbs (r>0.7, p<0.001). During the final stages, this was also evident in male animals. Locomotor activity was also correlated (r>0.6, p<0.001) with grip strength in the final stages of the disease.

### Brain growth arrest in *Hdh*Q150 mice is followed by a slowly progressive regional atrophy

**Fig 4:**
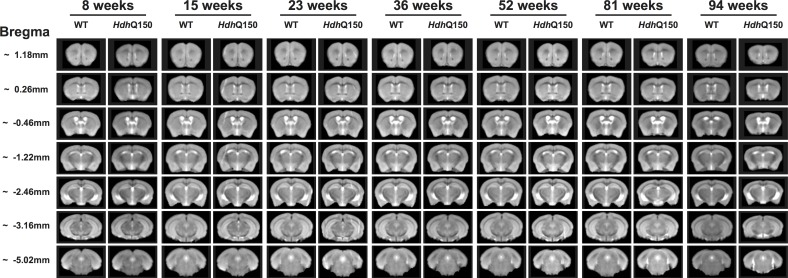
Longitudinal *in vivo* MR images. Summed T_2_-weighted structural group-images for WT and *Hdh*Q150 mice from 8 to 94 weeks of age.

**Fig 5:**
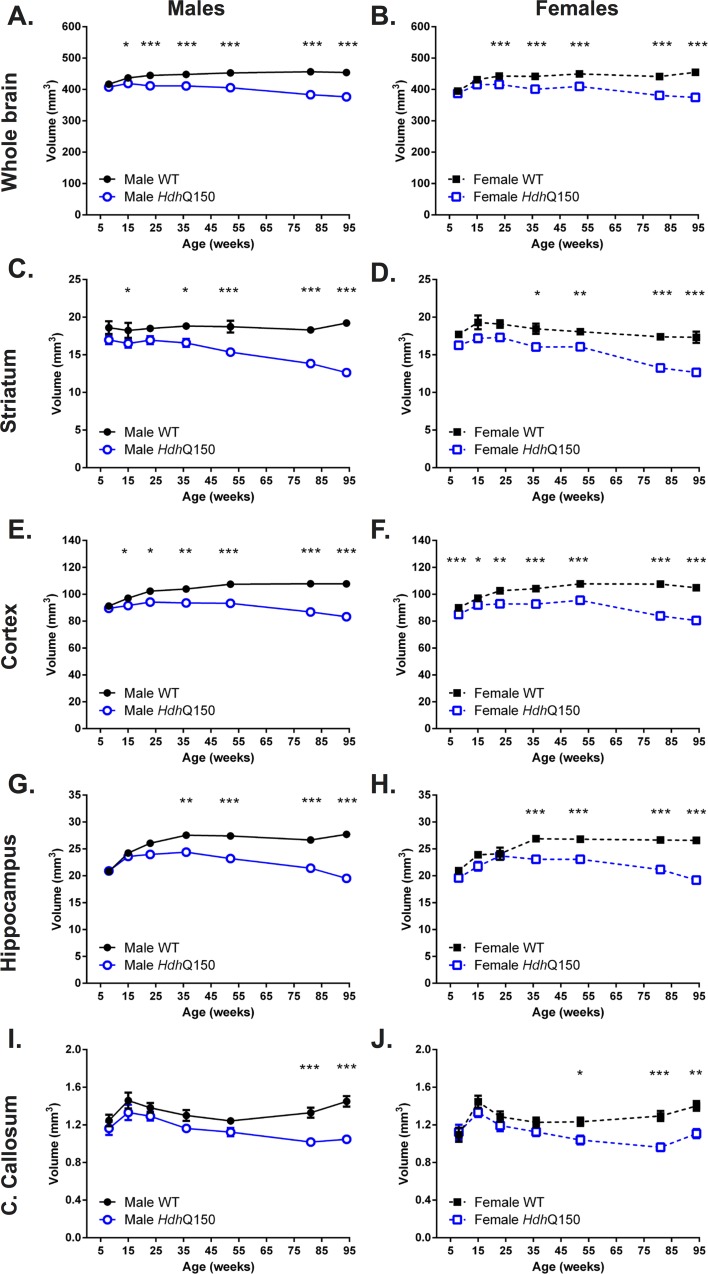
Quantification of regional brain atrophy. A gradual atrophy of whole brain atrophy was evident in male (A) and female (B) *Hdh*Q150 compared to controls. *Hdh*Q150 brain regions demonstrated progressive, age-related reduction in volume compared to WTs, irrespective of gender. Striatal volume loss in male *Hdh*Q150 (C) was greater than for females (D). This difference between male (E) and females (F) was also evident for the cortex. Although hippocampal volume differences were delayed compared to striatum and cortex, both male (G) and females (H) showed growth arrest and atrophy. Volumetric differences were also apparent in the corpus callosum with males (I) showing non-significantly lower volumes compared to females (J) with evidence of atrophy in both genders beyond 15 weeks of age. All data presented as means ± SEM; *p < .05, **p < .01, ***p < .001.

To provide a concomitant evaluation of brain anatomy, T_2_-weighted MR images were acquired that afforded a detailed assessment of changes in brain structure and tissue signal (Fig 4). A region-of interest (ROI) approach was used to measure volumes of anatomical structures (Fig 5). At 8 weeks of age, WT and *Hdh*Q150 animals showed equivalent whole brain volumes, but these revealed a slower growth up to 15 weeks of age and eventually growth arrest until 23 weeks of age (Fig 5A & 5B). After 23 weeks of age a very slow but progressive brain atrophy was observed. This global atrophy is driven by regional changes. Striatal volumes revealed significant differences at 15 weeks, with males exhibiting no growth until 23 weeks before progressive atrophy led to a 26% decrease in volume by 94 weeks (Fig 5C). This was the steepest decrease in regional volume. In females, striatal volume showed slower growth up to 15 weeks of age with no growth in volume up to 23 weeks of age (Fig 5D). Cortical volume for male (Fig 5E) and female *Hdh*Q150 (Fig 5F) revealed slower growth up to 23 weeks of age before a growth arrest period set-in that lasted until 52 weeks of age. Cortical atrophy was seen thereafter with 12% volume decrease leading to a 23% difference in volume with WT. The hippocampus (Fig 5G & 5H) saw a similar pattern of slower growth (8–23 weeks) followed by a period of stable volume (23–52 weeks) that eventual lead to shrinkage of tissue (21%). Interestingly, corpus callosum also saw a brief period of slower growth, but saw an early decrease in volume from 15 weeks onwards, potentially indicating important differences in connectivity that are not necessarily reflected in tissue volume changes (Fig 5I & 5J). Although there was little correlation between regional volumes in male and female WT mice, *Hdh*Q150 animals revealed a high degree of commonality in structural changes between regions (S4 Table). Striatal, cortical and hippocampal volume were all highly correlated with whole brain volume (r>0.6, p<0.001). Striatal volumes in *Hdh*Q150 were also correlated with hippocampal and cortical volumes, as well as that of the corpus callosum (r>0.4, p<0.001). However, corpus callosum was not correlated with cortical or hippocampal changes in *Hdh*Q150 animals.

Compaction of tissue is reflected in a T2 signal change (S5 Table). Over time there was a significant decrease in T2 signal in the striatum [F(Age) _1;246_ = 5.522, p<0.001], cortex [F(Age) _1;246_ = 8.194, p = 0.001], hippocampus [F(Age) _1;246_ = 4.785, p<0.001], corpus callosum [F(Age) _1;246_ = 30.229, p<0.001] and muscle [F(Age) _1;246_ = 19.762, p<0.001]. The T2 signal decreased in the striatum [F(Genotype X Age) _1;246_ = 2.572, p = 0.066] and hippocampus [F(Genotype X Age) _1;246_ = 2.345, p<0.05] as the animals grew older. The only overall genotype effect was in muscle [F(Genotype) _1;246_ = 4.714, p<0.05]. A gender effect was also evident in striatum [F(Gender) _1;246_ = 5.872, p<0.05], cortex [F(Gender) _1;246_ = 3.105, p = 0.079] and hippocampus [F(Gender) _1;246_ = 5.295, p<0.05]. Apart of a correlation between cortical and hippocampal T2 values (r>0.58, p<0.001), there were no associations between changes in T2 in any of the examined regions. These signal changes thus indicate the effect of aging on tissue, but also that the signal for *Hdh*Q150 animals evolves differently in these brain regions over time.

### Tensor-based morphometry dissociates global and regional atrophy

**Fig 6:**
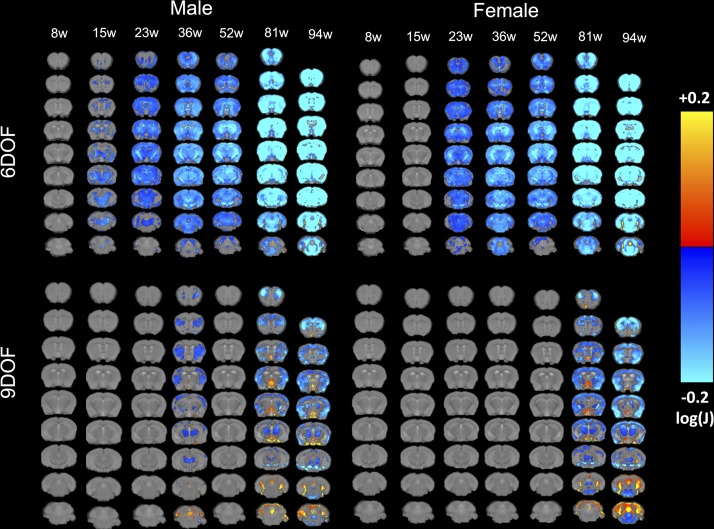
TBM Group comparison at each time point. Maps of local volumetric changes in male and female WT compared to *Hdh*Q150 mice revealed significant sub-regional changes. Images are presented either as global volume changes (6 degrees of freedom, DOF) or region-specific changes which account for whole brain differences (9DOF). Color scales represent statstically significant volume hypertrophy (warm colors) or atrophy (cold colors) with only voxels surviving correction for multiple comparisons (False Discovery Rate with q<0.05) being shown. Early atrophy in the striatum and motor cortex is evident before other regions are gradually affected. It is also evident here that 6DOF reveals more dramatic changes which potentially indicates overall brain growth related effect as compared to region-specific effects as revealed in the 9DOF.

To probe subtle sub-regional changes using an unbiased method that does not rely on predefined ROIs, tensor-based morphometry (TBM) visualized voxels that underwent statistically significant changes indicative of volumetric effects (Fig 6). A 6DOF registration provides a direct comparison between WT and *Hdh*Q150 indicating a gradual decrease in volume starting at 15 weeks. Subcortical areas were first affected, although the motor cortex revealed some atrophy early-on. To provide a direct regional comparison, that removes overall size effects, a 9DOF comparison was performed. This revealed that these early changes at 15 and 23 weeks of age were mostly driven by an overall size effect, as no direct regional effects were evident. In males, at 36 weeks regional effects were very localized to the motor and somatosensory cortices, but also subcortical structures, such as the striatum and thalamus. In this direct comparison, females only exhibited statistically significant changes at 81 weeks of age. These were akin to male subjects at this age, but with more widespread changes evident. In particular, there was an expansion of cortical atrophy and enlargement of ventricles. These results demonstrate that overall brain atrophy is a major driver in the disease process, although certain regions are more affected than others; these regional effects appear to be a secondary and potentially less important pathological event.

**Fig 7:**
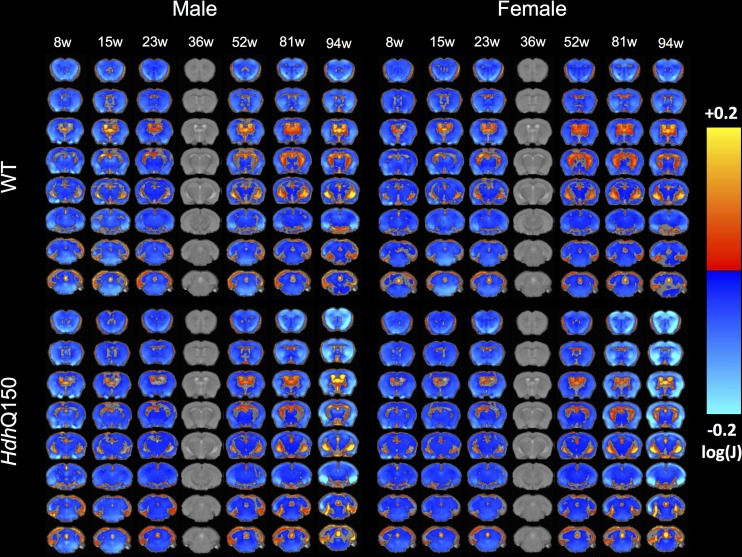
Age-related evolution of sub-regional changes in *Hdh*Q150 brains. To compare sub-regional changes over time, all MRI scans were co-registered to the 36 week time point as this represented the time point with the largest volume (9DOF) and the clinical horizon. This afforded a statistical comparison of tissue changes (decreases in cold colors, increases in warm colors) in either time direction for either WT or *Hdh*Q150 with a correction for multiple comparison (FDR with q<0.05). These results indicate a slower growth (cold colors) of striatum in *Hdh*Q150 animals with a greater decrease (cold colors) in striatum and cortical regions beyond the clinical horizon.

Due to the significant change in brain growth observed over the assessment period, a comparison across time that uses the first time point (8 weeks of age) as a baseline would be inappropriate. *Hdh*Q150 brains reached their largest size at 36 weeks, affording a visualizing of growth, as well as atrophy, in relation to this time point (Fig 7). As indicated by the ROI analysis, there was a slower brain growth between 8 and 36 weeks of age for *Hdh*Q150 animals of both genders (as indicated by the smaller decrease in volume at 8 weeks of age). Conversely, after 36 weeks of age a greater decrease in the cortex and striatum was observed in *Hdh*Q150 mice compared to WT, in contrast to a greater enlargement of the ventricles. Sub-cortical areas, such as the thalamus, appeared largely unaffected by the condition, although some decrease was observed at the final time point, potentially reflecting a general atrophy.

**Fig 8:**
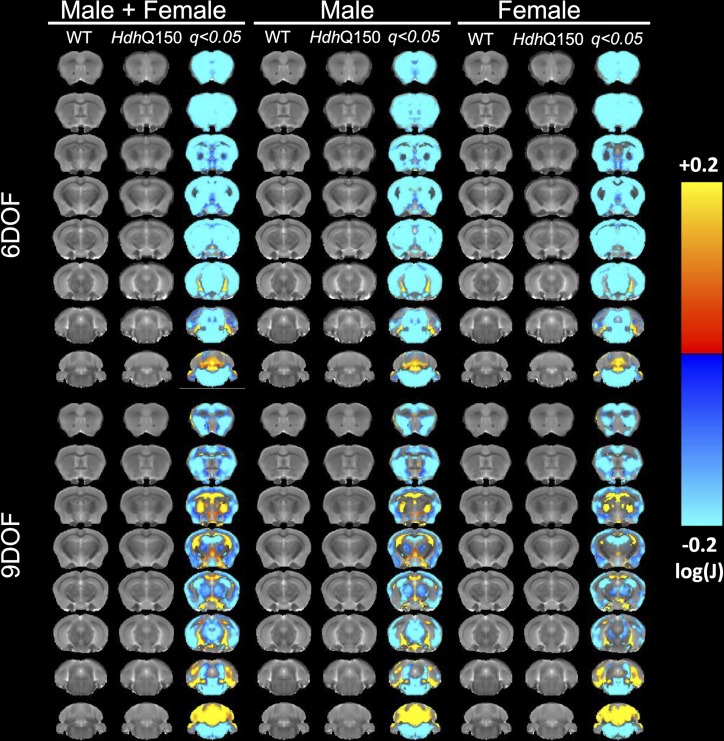
TBM on high resolution ex vivo T_2_-weighted MR images. To reduce potential partial volume effects in sub-regional comparisions *ex vivo* high resolution MR images were acquired to achieve a spatial resolution 2.5 higher than *in vivo*, as well as a >2x higher SNR, while avoiding motion related artefacts. This afforded a more precise investigation of subtle effects that might not be evident on *in vivo* images. A more defined sub-regional pattern of statstical differences between WT and *Hdh*Q150 was evidence using this approach. Color scales indicate statstically significant volume decreases (cold colors) and increases (warm colors) with correction for multiple comparisons (FDR with q<0.05).

*Ex vivo* MRI scans provided a higher spatial resolution with improved signal-to-noise ratio in the absence of physiological motion allowing a more detailed sub-regional comparison of WT with *Hdh*Q150 mice (Fig 8). However, 9DOF are required for these subtle regional nuances to be apparent, with 6DOF only revealing a blanket decrease in almost all structures. Using 9DOF, sub-regional changes, notably within the cortex, become apparent with the outer layers undergoing greater loss. The motor and somatosensory regions showed a greater extent of atrophy than the enthorhinal cortex. In contrast, the striatum was uniformly decreased. The septal areas also appeared to be affected, but it is possible that these were spill-over effects due to the decreased volume in the striatum. A very subtle decrease in the thalamic area was also becoming apparent. The ventricles and adjacent areas (such as the hippocampus) revealed increases. These changes were fairly consistent across both genders although minor differences were evident especially in regions with subtle changes.

### Only late stage brain atrophy correlates with behavioral impairments

To investigate how these structural changes relate to the behavioral performance of WT and *Hdh*Q150 mice, correlations were performed between behavioral and MRI measures (S6 Table). After correcting for multiple comparisons, only a few random correlations emerged up to 49–54 weeks of age. At this stage, a pattern emerged with grip strength of all paws being correlated significantly with atrophy of the whole brain, striatum, cortex and hippocampus (all r>0.7, p<0.001), but not corpus callosum. No correlation with T2 signal intensity was evident at this time point. After 79 weeks of age this correlation also extended to the forepaw only grip strength (all r>0.7, p<0.001), but not for hippocampus (r = 0.68, n.s.). Locomotor activity showed the same correlations with whole and regional brain volumes, including hippocampus (all r>0.7, p<0.001), except corpus callosum (r = 0.677, n.s.). T2 signal intensity measures essentially revealed the same pattern of correlations as volume changes with behavioral performance at these later stages of the disease. These later-stage correlations were expected considering that behavioral performance and MRI measures by themselves are highly correlated, indicating a common path for all measures [30, 31].

### Neurodegeneration and mHTT in striatum and cortex

**Fig 9:**
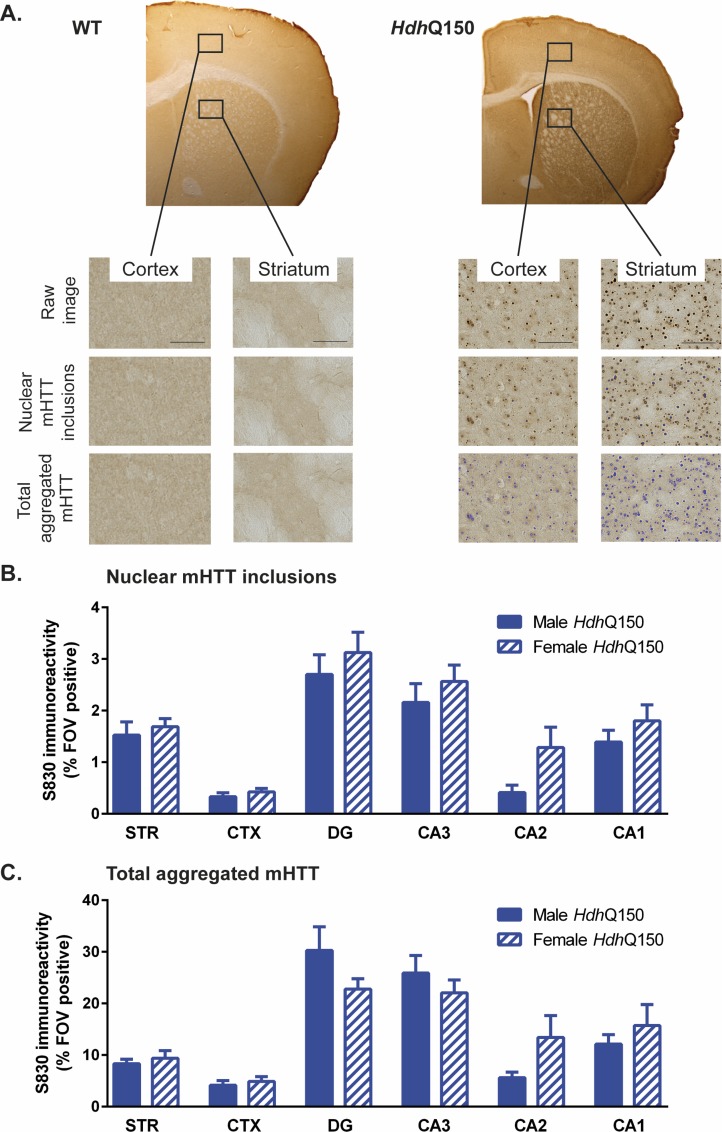
mHTT accumulation in *Hdh*Q150 mice. (A) Sample S830-stained brain sections of a WT and *Hdh*Q150 mouse. Threshold-intensity-based analysis was used to descriminate nuclear mHTT inclusions versus total aggregated mHTT levels in the striatum (STR), cortex (CTX), dentate gyrus (DG), hippocampal CA3 subfield (CA3), hippocampal CA2 subfield (CA2) and hippocampal CA1 subfield (CA1). There were no significant differences in mHTT levels between male and female *Hdh*Q150 mice in the regions investigated. All data presented as means ± SEM.

To investigate the underlying molecular and cellular changes of this anatomical atrophy, histopathological studies investigated the distribution of aggregated mHTT, as well as neuronal loss. The presence of aggregated mHTT within different regions was revealed by immunohistochemistry (Fig 9A). Given that mHTT is absent in WT mice, it was only quantified in *Hdh*Q150 animals to provide a region and gender comparison. The presence of nuclear mHTT inclusions was consistent with regions that contained a high neuronal density, such as the hippocampal subfields (Fig 9B). The nuclear inclusion load was also higher in striatal cells than in the cortex. Although there was no significant difference between males and females, females tended to have more nuclear inclusions than males. These general regional differences were also evident for total aggregated mHTT (Fig 9C) with no significant gender effect. However, compared to nuclear mHTT, total aggregated mHTT showed lower levels in the dentate gyrus and CA3, whereas the nuclear content was higher for females in these regions compared to male *Hdh*Q150 mice. Interestingly total aggregated mHTT load in the striatum was only highly correlated with CA1 (r = 0.919, p<0.001) and CA2 (r = 0.837, p<0.001), whereas nuclear mHTT revealed correlations of cortex with striatum (r = 0.777, p<0.001), dentate gyrus (r = 0.961, p<0.001) and CA3 (r = 0.846, p<0.001) (S7 Table).

**Fig 10:**
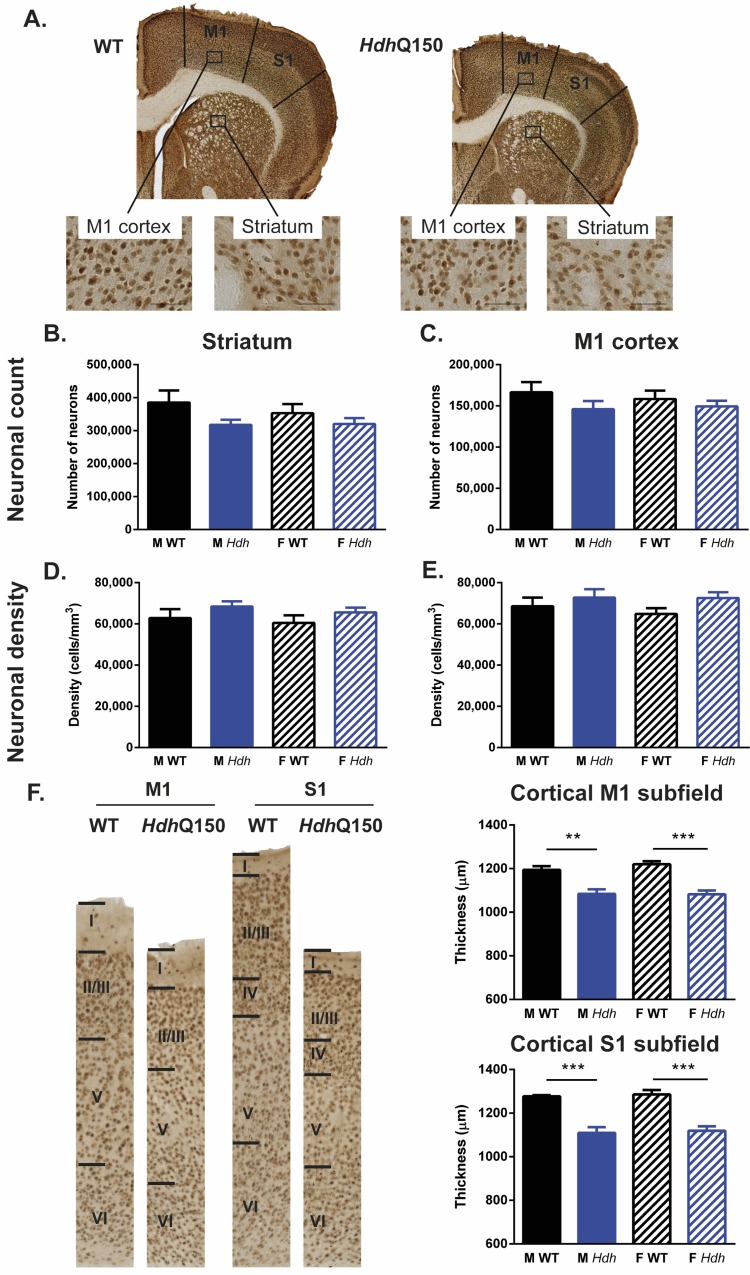
Histological analyses of NeuN-stained sections. (A) Sample NeuN-stained brain sections of a WT and *Hdh*Q150 mouse. Although *Hdh*Q150 mouse neuronal numbers were consistently lower than those of their WT controls, there was no significant difference in neuronal counts in the striatum (B) or M1 cortex (C). Similarly, despite consitently higher neuronal density levels in *Hdh*Q150s, there was no significant difference compared to WTs in striatum (B) or M1 cortex (E). There was substantial M1 and S1 cortical thinning in *Hdh*Q150 mice (F).

To determine if neurodegeneration was a major determinant of atrophy in the striatum and cortex, neuronal number and density were quantified using stereological methods (Fig 10A). At 94 weeks of age, there was a 18% decrease in neurons in the striatum (Fig 10B) of male *Hdh*Q150 mice, but the genotype comparison did not reach the criterion for significance [F(Genotype) _1;34_ = 4.016, p = 0.054]. There was also no statistically significant decrease in the M1 cortex for both genders [F(Genotype) _1;34_ = 2.249, n.s], with a 14% decrease for male *Hdh*Q150 and 13% decrease for females (Fig 10C). Neuronal density was slightly increased (9%) for *Hdh*Q150 animals in the striatum (Fig 10D), but did not reach statistical significance [F(Genotype) _1;34_ = 2.572, n.s]. Likewise, in the cortex, a 11% increase was evident, but did not reach significance (Fig 10E) [F(Genotype) _1;34_ = 2.928, p = 0.097]. However, an increase in neuronal density and loss did translate into atrophy of the cortex (Fig 10F) and this was evidenced by a significant decrease in M1 [F(Genotype) _11034_ = 49.276, p<0.001] and S1 cortical thickness [F(Genotype) _1;34_ = 59.446, p<0.001]. None of these histological measures revealed a gender effect. However, a correlational analysis revealed a gender effect with female WT and *Hdh*Q150 M1 neuronal counts (r = 0.699, p<0.001), density (r = 0.683, p<0.001) and volume (r = 0.673, p<0.001) correlating with their striatal counterparts (S8 Table). Although in males there were also correlations (r>0.5, n.s.), these did not reach statistical significance. In contrast in males, statistical significant correlations were found between the number of striatal neurons and volume (r = 0.745, p<0.001). In females this correlation (r = 0.526, n.s.) was not significant after multiple comparison correction.

### Histopathological measures are poor predictors of behavior or MRI measures

To determine if there was an association between histopathological measures and behavioral changes, a correlational analysis was performed. No significant correlations were evident between mHTT levels and behavioral measures in *Hdh*Q150 animals (S9 Table). When both genders were combined for WT or *Hdh*Q150 significant correlations (S10 Table) were evident for grip strength forepaws (males r = 0.672, p<0.001), as well as all four paws with striatal volume (males r = 0.711, p<0.001; females r = 0.834, p<0.001). M1 cortical volume in males also correlated with forepaw grip strength (r = 0.504, p<0.001). In females, a strong correlation was also apparent (r = 0.585, ns), but this was not statistically significant after correction for multiple comparisons. In summary, a robust and strong association between histopathological measure and behavioral changes was not identified.

A general lack of correlations was also evident for mHTT and MRI measures (S11 Table), with the only exception being a very strong significant negative correlation between nuclear inclusion levels in the hippocampal CA3 region and T2 relaxivity in the cortex (r = -0.958, p<0.001) of males. Cortical relaxivity also correlated with nuclear inclusions in all other regions (r>0.5), but these did not reach statistical significance after correcting for multiple comparisons. Females did not show these high levels of correlation. Total aggregated mHTT in general exhibited weak correlations with MRI measures (r<0.3). T2 relaxivity also did not reveal any correlation with neuronal measures (S12 Table). For the combined WT and *Hdh*Q150, significant correlations were present for striatal volume by histology and whole brain (male r = 0.761, p<0.001; female r = 0.718, p<0.001) and hippocampal volume by MRI (male r = 0.738, p<0.001; female r = 0.697, p<0.001). In females, histology-based striatal volume was also significantly correlated with MR-based striatal (r = 0.712, p<0.001) and cortical volumes (r = 0.686, p<0.001). In males, these correlations did not survive the correction for multiple comparisons. There were no correlations for neuronal density or neuronal numbers with MRI measures. The lack of correlations between mHTT aggregation and neuron numbers with MRI measures further indicates that other factors contribute to macroscopic tissue changes in Huntington’s disease.

## Discussion

Establishing the neurobiological cascade of pathological changes and identifying pre-manifest biomarkers is highly dependent on the use of animal models of HD. We here characterized the *Hdh*Q150 knock-in model of HD to reveal a delayed onset of motor signs accompanied by regional volumetric brain changes. The behavioral manifestation occurred around 36 weeks of age, with pre-manifest changes in striatum, cortex and hippocampus evident. At 94 weeks (2 years of age), *Hdh*Q150 exhibited widespread mHTT deposits. However, there was no strong correlation between behavioral, MRI and neuropathological measures. Although these measures all decreased with time and it is tempting to associate these changes with each other, the lack of a correlation between these implicate additional factors. This characterization indicates that the *Hdh*Q150 model is useful to identify and further investigate pre-manifest biomarkers, but that there is still an insufficient understanding of the neurobiological cascade to link mHTT deposits with the emergence of a clinical horizon.

### *Hdh*Q150 mice exhibit a slowly progressive brain atrophy with motor impairment

The key features of Huntington disease are a slowly progressive neurodegeneration accompanied by motor impairments, as well as the emergence of cognitive deficits [3]. The *Hdh*Q150 knock-in model revealed motor deficits that are evident at 36 weeks of age (9 months) with a slowly progressive phenotype. However, grip strength of forepaws indicated even earlier deficits occurring in male *Hdh*Q150 animals at 9 weeks of age, although a decline in strength only becomes progressive after 38 weeks of age, which is consistent with a previous report of motor dysfunction in this model [16]. However, this motor dysfunction was only evident in behavioral tasks that did not rely on balance, with an increase in weight and size in WT animals producing a poor performance on the rotarod test. Although we did not find any cognitive dysfunction in the test battery used here, impairments in the water maze and prepulse inhibition have been reported as early as 3–4 months of age [16]. The “clinical horizon” of *Hdh*Q150 animals therefore manifests itself at approximately 36 weeks (6 months) of age. Interestingly, this is also the time point where the most significant shift in brain structures was evident.

Prior to 36 weeks of age, the growth of most brain regions was slower for the *Hdh*Q150 mice, whereas beyond this point, a progressive atrophy of the structures became apparent. Growth of the striatum was already stymied at 15 weeks of age. Although female *Hdh*Q150 still demonstrated some growth until 15 weeks of age, this was not the case for males, potentially indicating a difference in pathology that can explain the more severe behavioral phenotype observed in males. Nevertheless, other regions, such as cortex and hippocampus underwent a prolonged slow growth up to 23 weeks, with no further growth between 23–36 weeks. This period could be a tipping point, where slow growth and atrophy reach equilibrium. It is conceivable that neurodegeneration occurs throughout development and this underlies the slow growth observed here. The slow regional brain growth in WT mice beyond 36 weeks suggests that the progressive decline observed in *Hdh*Q150 is no longer compensated by growth. The slow growth in the striatum and cortex observed in *Hdh*Q150 might actually reflect neurodegeneration, which is masked by concurrent growth. In human studies, no extensive longitudinal MRI studies from pre-manifest to late stage disease have been reported. A smaller basal ganglia volume is generally considered atrophy due to a cross-sectional comparison [51, 54], but serial measurements for 2 years in pre-manifest patients potentially suggest compensatory anatomical changes preceding the manifestation of HD [55].

The TBM comparisons revealed that sub-regional changes were more evident with male *Hdh*Q150 animals, as evidenced by a smaller striatum, thalamus and motor cortex at 15 weeks of age. These volume changes were exacerbated with time in terms of regional distribution, as well as magnitude. As the structural changes in the striatum became more pronounced, atrophy in the cortex also became apparent. This spatiotemporal evolution progressed to a point whereby the regional atrophy did not progress further. Indeed, whole brain volume showed a decrease between 52 and 81 weeks, but then leveled off between 81–94 weeks. Females showed the same pattern of spatiotemporal evolution, but its onset was delayed. Nevertheless, at 94 weeks both male and female *Hdh*Q150 displayed a very similar pattern and magnitude of change. In females, the decline observed after growth arrest was greater than in males. Although estrogen has been suggested to act a neuroprotective agent [56], the rapid decline of the striatum and cortex in females indicates that the later onset in atrophy is more likely due to a stronger compensatory growth in these animals, rather than a reduction in neurodegeneration. Although there is evidence here of significant neuronal loss in striatum and cortex at 94 weeks, it is unclear if indeed neuronal loss is observed in the pre-manifest stage (i.e. <36 weeks) or if the volumetric difference is due to tissue compaction, as observed in the R6/2 model at 14 weeks of age [30]. Indeed, nuclear inclusions are densely distributed at 5 months of age [17], with gene expression changes being mostly related to chromatin organization at 6 months and intracellular signaling at 18 months [26].

### Contrasting N-terminal fragment with full-length knock-in models

R6/2 mice exhibit a rapidly progressing phenotype with a very significant motor dysfunction and premature death [57]. There is also evidence that the striatum does not exhibit a normal growth pattern, with no further increase in volume beyond 4 weeks of age. Cortex exhibits a significant decrease in volume after 4 weeks of age. As the R6/2 at 14 weeks of age do not exhibit actual neuronal loss [30], this further suggests that initial regional changes in the brain are not reflective of neurodegeneration *per se*, but other tissue changes, such as increased neuronal density and reduction in extracellular space. Indeed, R6/1 mice that live longer than R6/2 exhibit neuronal loss at 19 weeks of age [30, 31]. Although these two mouse lines are more aggressive in their behavioral phenotype, including premature death, they nevertheless indicate that early brain tissue changes are not necessarily linked to a classical loss of neurons, but other changes precede this phenomenon [18, 20]. In *Hdh*Q150 animals, the prolonged pre-manifest phase that is characterized by thwarted regional growth is likely to reflect similar changes.

The robust and severe behavioral phenotype of motor and cognitive deficits in the R6/2 [30] and R6/1 model [31] contrasts with the milder signs observed in *Hdh*Q150. The lack of correlation with structural changes, even early pre-manifest changes apparent on anatomical T_2_-weighted scans, suggest that cellular dysfunction precedes neuronal loss [18, 26], but that other tissue changes that are reflected in gross morphological anatomical differences precede these functional deficits. Expression of mHTT in specific cellular phenotypes, such as astrocytes and neurons, can help to address this issue [58]. Insufficient clearing of excitotoxic glutamate by compromised astrocytes [59, 60], as well as a different reactivity, could all be contributing factors. Moreover, there is a general lack of consideration of the extracellular space (20% of total brain volume), which at least in R6/2 is reduced [30], given a decrease in tissue volume in the absence of neuronal loss leading to an increase in neuronal density.

The consistent characterization of different HD models using the same methodology allows us a direct comparison of how differences in introducing mutant HTT affects the emergence and presentation of molecular pathology and the causal cascade that ensues [14]. The lower transgene expression levels in R6/1 animals compared to R6/2 afforded a longer lifespan due to a milder phenotype that was evident in a slower progression of the condition in terms of behavioral deficits, as well as brain atrophy [30, 31]. Importantly, this slower phenotype in R6/1 produced neuronal loss that was not evident in R6/2. Still, there remains a disconnect between gross anatomical changes, as well as neuronal loss, and behavioral dysfunction [2]. This was also the case here in the *Hdh*Q150, which had a similar repeat length to R6/2, but survived significantly longer with a phenotype developing much more slowly, reaching a clinical horizon at 36 weeks rather than 4–6 weeks in R6/2 mice. The R6/2 and R6/1 mouse models are therefore best suited to “high throughput” exploratory in vivo experiments [57], whereas the *Hdh*Q150 mouse model of HD is more adequate to monitor pre-manifest changes due to its prolong period leading up to the clinical horizon [14]. The *Hdh*Q150 model also has greater face validity to identify targets and evaluate interventions due to the slower pathological evolution [9, 32]. Especially targets, such as astrocytosis or microglia response, can be expected to be quite different in rapidly versus more slowly evolving neurodegeneration paradigms [61, 62].

### Challenges to establish pre-manifest markers in animal models of HD

The extensive pre-manifest period in *Hdh*Q150 mice provides a unique setting to probe biomarkers that can be used to monitor the progression of subjects towards the clinical horizon and potentially serve as surrogates to evaluate pre-symptomatic interventions. MRI here demonstrated measurable differences between *Hdh*Q150 and WT animals. Notably, striatal volume was reduced even at 8 weeks of age. By 15 weeks of age, motor cortex and striatum were reduced, with more widespread changes evident at 23 weeks prior to the emergence of behavioral deficits at 36 weeks of age. The onset of behavioral deficits also coincided with a shift from brain regions not growing, to an actual decline. These subtle changes can be identified in the *Hdh*Q150 model with disease load being well controlled. In patients, these changes would be more difficult to identify. Nevertheless, there is evidence that caudate volume is smaller in pre-manifest patients [63], potentially highlighting the value of the *Hdh*Q150 to further develop MRI-based biomarkers. Although whole brain atrophy has also been suggested as a pre-manifest measure [64], more defined regional changes, such as in the caudate, might provide earlier and more specific biomarkers to monitor the disease time course [65].

A major challenge in patients, as in animal models, is the use of longitudinal MRI and to choose appropriate time points [54]. As it is unknown in patients when the clinical horizon arises, investigations into the pre-manifest phase are difficult in terms of their timing, as well as longitudinal follow-up [66]. Most MRI-based biomarker studies therefore rely on cross-sectional images, although a few studies have used longitudinal serial MRI scans [54]. Studies in transgenic animals provide a level of control that cannot be achieved in patients and can serve to identify, as well as to validate, MRI-based biomarkers [9, 67]. Being able to perform time-matched concomitant behavioral analyses further affords an investigation of how low-level changes in anatomy eventually cumulate into behavioral manifestations, which will be difficult to achieve in patients considering their heterogeneity and difficulty in predicting a disease time course. A further advantage of animal studies is the potential to provide histological and molecular analyses, which are unlikely to be available in pre-manifest patients. The *Hdh*Q150 model provides an extended pre-manifest period that facilitates more extensive studies on this period. Investigations that go beyond simple structural measures, as used here, should be performed. Ideally, MR spectroscopy and diffusion MRI complement T_2_-weighted structural scans, as these can potentially provide more specific markers of tissue changes in the striatum and cortex [68, 69]. It is important to note though that even in transgenic animals these investigations are not trivial in terms of logistics and costs, but could provide robustly quantifiable and validated biomarkers to define the various stages of the condition.

In animal studies commonly the aim is to keep the CAG repeat length consistent across experimental groups to ensure low variability in outcome measures to increase statistical power and detect significant effects between groups. This allowed us here to use only ~10 subjects per condition to find significant differences between WT and *Hdh*Q150. However, almost no correlations between measures survived correction for multiple comparisons, although there are significant reports in clinical cohorts associating structural MRI changes with behavioral measures. The key difference here is that the load of CAG repeats in clinical trials is almost always very variable. This leads to subjects with high and low CAG repeats being included in the correlation analysis, whereas in our preclinical study the variability of CAG was minimal. Our correlations here therefore reflect more directly the relationship between MRI measures and behavior, whereas clinically CAG repeat loading is a significant co-variate (i.e. disease load effect on measures), which might determine this relationship as a common hidden variable. Indeed, there is extensive evidence in clinical studies that CAG repeat length is associated with brain atrophy and clinical signs [65]. Specifically designed and controlled animal studies (i.e. low versus high CAG variability) are required to unravel these relationships.

## Conclusions

This study performed longitudinal serial MRI scans with concomitant behavioral assessments of motor and cognitive tasks over a 94 week time span in the *Hdh*Q150 knock-in model of HD. Longitudinal studies of anatomy, as well as behavior, are key to characterize the phenotype of these animals. We here demonstrated that unlike the R6/2 and R6/1 transgenic mouse models, a prolonged pre-manifest period of 36 weeks is present in *Hdh*Q150 animals that affords the identification of MRI-based biomarkers, notably reduced striatal and cortical volumes. However, there were few significant correlations between outcome measures potentially indicating that other tissue measures, such as astrocytes, might be important in the causal cascade between mHTT aggregation and the emergence of behavioral deficits. The *Hdh*Q150 mouse model of Huntington’s disease presents an excellent system to identify novel biomarkers, as well as their utility in identifying interventions that act on the pre-manifest stage of the condition.

## Supporting Information

S1 Table Number of animals used. All tests were conducted on the same cohort of animals. Nevertheless, animals used for each comparison varied due to either death during the study, missing data due to non-performance of test, or exclusion of statistical outliers.
(TIFF)

S2 Table Main effects derived from statistical analyses. Main effects derived from two- and three-way ANOVAs (F values followed by degrees of freedom in subscript parentheses). LMA = locomotor activity in an open-field, GS FL = fore limb grip strength, GS 4L = fore and hind limb grip strength, TM CL = cued learning in a swimming T-maze, TM CR = cue reversal learning in a swimming T-maze, OD = odor descrimination, SI = social interaction, WB = whole brain, STR = striatum, CTX = cortex, HIPP = hippocampus, CC = corpus callosum, MUSC = cheek muscle, M1 = M1 cortex, S1 = S1 cortex.
(TIFF)

S3 Table Correlations of behavioral measures. Correlations of performance at behavioral tasks collected at six timepoints (9–13 weeks, 16–21 weeks, 26–27 weeks, 34–39 weeks, 49–54 weeks, 79–80 weeks) presented as Pearson r values. GS FL = fore limb grip strength, GS 4L = fore and hind limb grip strength, LMA = locomotor activity in an open-field, TM CL = cued learning in a swimming T-maze, TM CR = cue reversal learning in a swimming T-maze, OD = odor discrimination, SI = social interaction. *Statistically significant after Bonferroni Correction (adjusted p value: 9–13 weeks p = 0.0033; 16–21 weeks p = 0.0024; 26–27 weeks p = 0.0083; 34–39 weeks p = 0.0024; 49–54 p = 0.0024; 79–80 weeks p = 0.005; 92–94 weeks p = 0.0083).
(TIFF)

S4 Table Correlations of *in vivo* MRI measures. Correlations of MR measure of pathology over time presented as Pearson r values. WB = whole brain, STR = striatum, CTX = cortex, HIPP = hippocampus, CC = corpus callosum, MUSC = cheek muscle. *Statistically significant after Bonferroni Correction (adjusted p value 0.001).
(TIFF)

S5 Table Regional T2 relaxivity. Mean (SEM) T2 relaxation times (msec) for four brain regions and cheek muscle tissue across the seven *in vivo* MRI scans.
(TIFF)

S6 Table Correlations of behavioral versus MRI measure of pathology. Correlations of behavioral measures against age-matched MRI measures at six time points (8–13 weeks, 15–21 weeks, 23–27 weeks, 34–39 weeks, 49–54 weeks, 79–81 weeks, 93–94 weeks) presented as Pearson r values. GS FL = fore limb grip strength, GS 4L = fore and hind limb grip strength, LMA = locomotor activity in an open-field, TM CL = cued learning in a swimming T-maze, TM CR = cue reversal learning in a swimming T-maze, OD = odor descrimination, SI = social interaction, WB = whole brain, STR = striatum, CTX = cortex, HIPP = hippocampus, CC = corpus callosum, MUSC = cheek muscle. *Statistically significant after Bonferroni Correction (adjusted p value: 8–13 weeks p = 0.0017; 15–21 weeks p = 0.0014; 23–27 weeks p = 0.0025; 34–39 weeks p = 0.0014; 49–54 weeks p = 0.0014; 79–81 weeks p = 0.002; 93–94 weeks p = 0.0025).
(TIFF)

S7 Table Correlation of regional mHTT levels. Correlations of total aggregated mHTT (Total mHTT) and nuclear mHTT inclusions (Nuc mHTT) measured on S830-stained sections, presented as Pearson r values. STR = striatum, CTX = cortex, DG = hippocampal dentate gyrus, CA1 = hippocampal CA1 subfield, CA2 = hippocampal CA2 subfield, CA3 = hippocampal CA3 subfield. *Statistically significant after Bonferroni Correction (adjusted p value 0.0008).
(TIFF)

S8 Table Correlation of neuronal characteristics. Correlation of stereological measures of neuronal characteristics on NeuN-stained brain sections, presented as Pearson r values. STR = striatum, M1 CTX = M1 cortex, Neur no. = neuronal number, Neur dens. = neuronal density. *Statistically significant after Bonferroni Correction (adjusted p value 0.003).
(TIFF)

S9 Table Correlation of mHTT levels and behavioral performance. Correlations of total aggregated mHTT (Total mHTT) and nuclear mHTT inclusions (Nuc mHTT) versus behavioral performance at the final time point (92–94 weeks). STR = striatum, CTX = cortex, DG = hippocampal dentate gyrus, CA1 = hippocampal CA1 subfield, CA2 = hippocampal CA2 subfield, CA3 = hippocampal CA3 subfield, GS FL = fore limb grip strength, GS 4L = fore and hind limb grip strength, LMA = locomotor activity in an open-field, OD = odor descrimination. There were no significant correlations after Bonferroni Correction (adjusted p volue 0.002).
(TIFF)

S10 Table Correlation of neuronal characteristics and behavioral performance. Correlations of stereological measures of neuronal characteristics versus behavioral performance at the final time point (92–94 weeks). STR = striatum, M1 CTX = M1 cortex, Neur no. = neuronal number, Neur dens. = neuronal density, GS FL = fore limb grip strength, GS 4L = fore and hind limb grip strength, LMA = locomotor activity in an open-field, OD = odor descrimination. *Statistically significant after Bonferroni Correction (adjusted p value 0.0042).
(TIFF)

S11 Table Correlation of mHTT levels and MRI measures of pathology. Correlations of total aggregated mHTT (Total mHTT) and nuclear mHTT inclusions (Nuc mHTT) versus MRI measures taken at the final *in vivo* time point (94 weeks). STR = striatum, CTX = cortex, DG = hippocampal dentate gyrus, CA1 = hippocampal CA1 subfield, CA2 = hippocampal CA2 subfield, CA3 = hippocampal CA3 subfield, WB = whole brain, HIPP = hippocampus, CC = corpus callosum, MUSC = cheek muscle. *Statistically significant after Bonferroni Correction (adjusted p value 0.0017).
(TIFF)

S12 Table Correlation of neuronal characteristics and MRI measures. Correlations of stereological measures of neuronal characteristics versus MRI measure of pathology taken at the final *in vivo* time point (94 weeks), presented as Pearson r values. STR = striatum, M1 CTX = M1 cortex, Neur no. = neuronal number, Neur dens. = neuronal density, WB = whole brain, CTX = cortex, HIPP = hippocampus, CC = corpus callosum, MUSC = cheek muscle. *Statistically significant after Bonferroni Correction (adjusted p value 0.003).
(TIFF)
