# Supplementary figures and images for: Correlations of Behavioral Deficits with Brain Pathology Assessed through Longitudinal MRI and Histopathology in the HdhQ150/Q150 Mouse Model of Huntington’s Disease
Source: PLoS One. 2017 Jan 18;12(1):e0168556. doi: 10.1371/journal.pone.0168556 (PMC5242535; doi:10.1371/journal.pone.0168556)

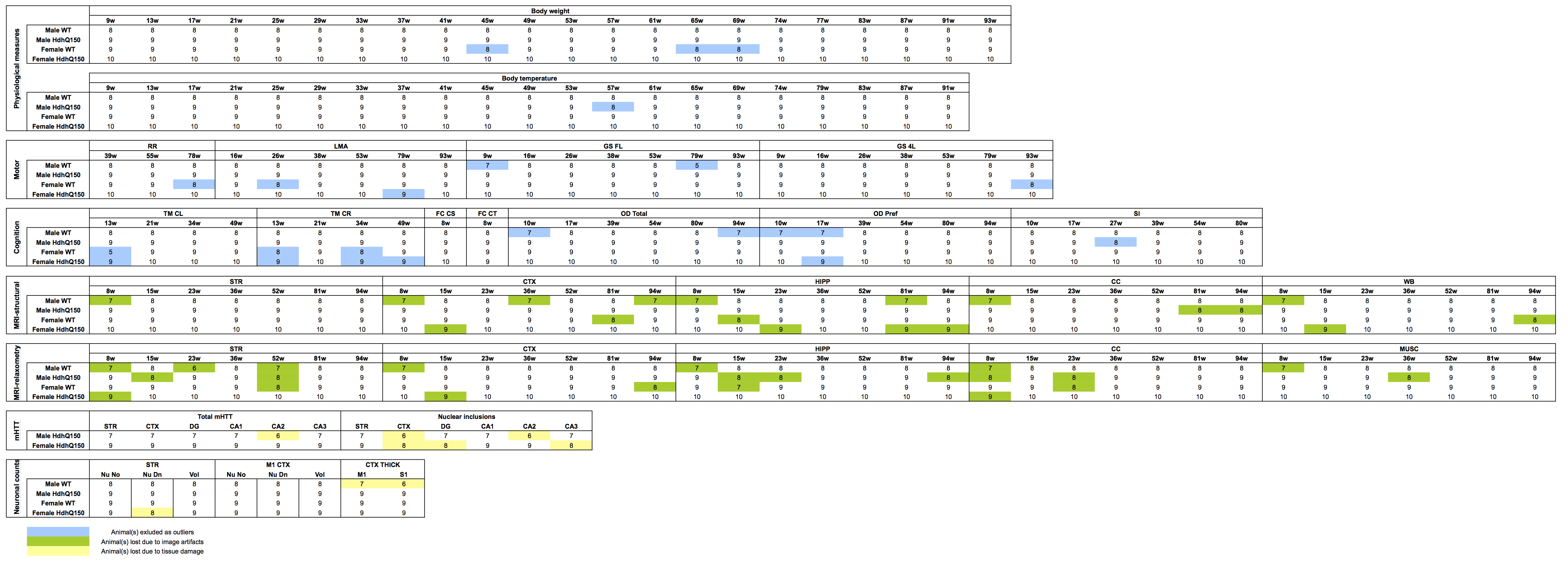

Supplement: S1 Table — All tests were conducted on the same cohort of animals. Nevertheless, animals used for each comparison varied due to either death during the study, missing data due to non-performance of test, or exclusion of statistical outliers. (TIFF) [file pone.0168556.s001.tiff]

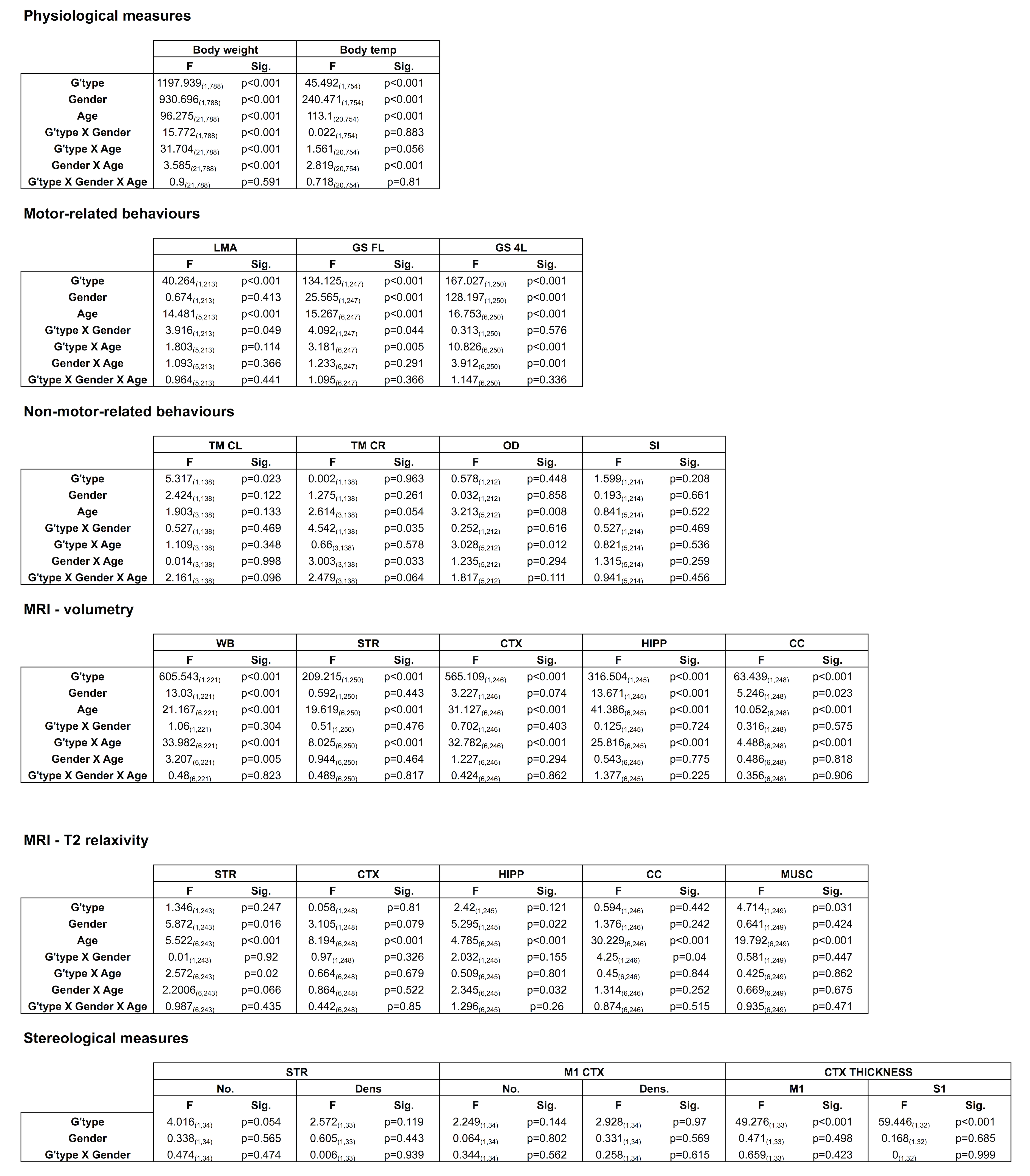

Supplement: S2 Table — Main effects derived from two- and three-way ANOVAs (F values followed by degrees of freedom in subscript parentheses). LMA = locomotor activity in an open-field, GS FL = fore limb grip strength, GS 4L = fore and hind limb grip strength, TM CL = cued learning in a swimming T-maze, TM CR = cue reversal learning in a swimming T-maze, OD = odor descrimination, SI = social interaction, WB = whole brain, STR = striatum, CTX = cortex, HIPP = hippocampus, CC = corpus callosum, MUSC = cheek muscle, M1 = M1 cortex, S1 = S1 cortex. (TIFF) [file pone.0168556.s002.tiff]

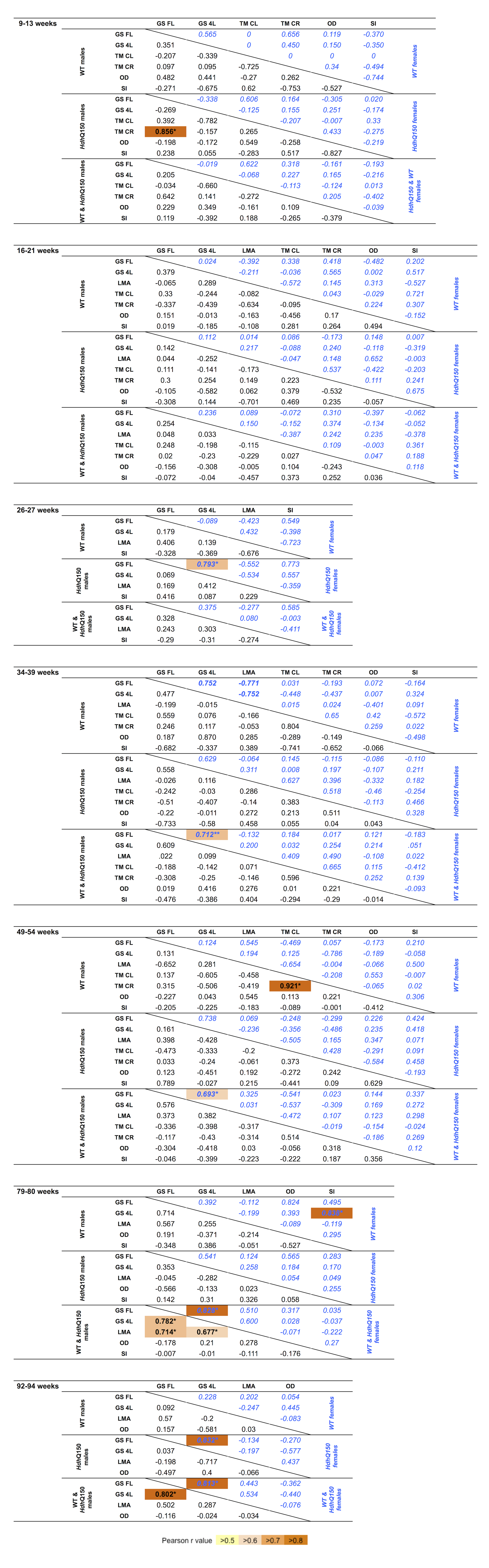

Supplement: S3 Table — Correlations of performance at behavioral tasks collected at six timepoints (9–13 weeks, 16–21 weeks, 26–27 weeks, 34–39 weeks, 49–54 weeks, 79–80 weeks) presented as Pearson r values. GS FL = fore limb grip strength, GS 4L = fore and hind limb grip strength, LMA = locomotor activity in an open-field, TM CL = cued learning in a swimming T-maze, TM CR = cue reversal learning in a swimming T-maze, OD = odor discrimination, SI = social interaction. *Statistically significant after Bonferroni Correction (adjusted p value: 9–13 weeks p = 0.0033; 16–21 weeks p = 0.0024; 26–27 weeks p = 0.0083; 34–39 weeks p = 0.0024; 49–54 p = 0.0024; 79–80 weeks p = 0.005; 92–94 weeks p = 0.0083). (TIFF) [file pone.0168556.s003.tiff]

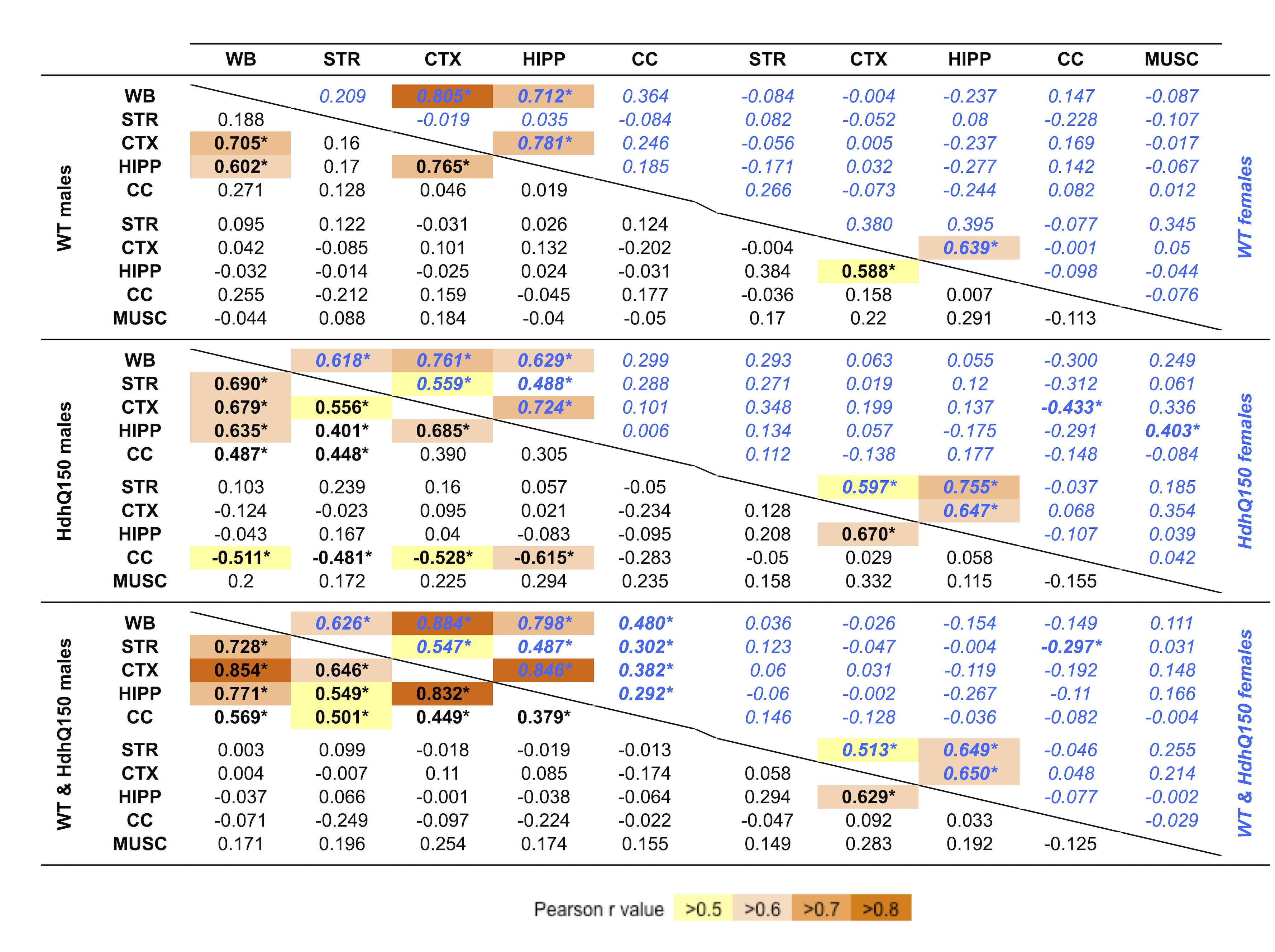

Supplement: S4 Table — Correlations of MR measure of pathology over time presented as Pearson r values. WB = whole brain, STR = striatum, CTX = cortex, HIPP = hippocampus, CC = corpus callosum, MUSC = cheek muscle. *Statistically significant after Bonferroni Correction (adjusted p value 0.001). (TIFF) [file pone.0168556.s004.tiff]

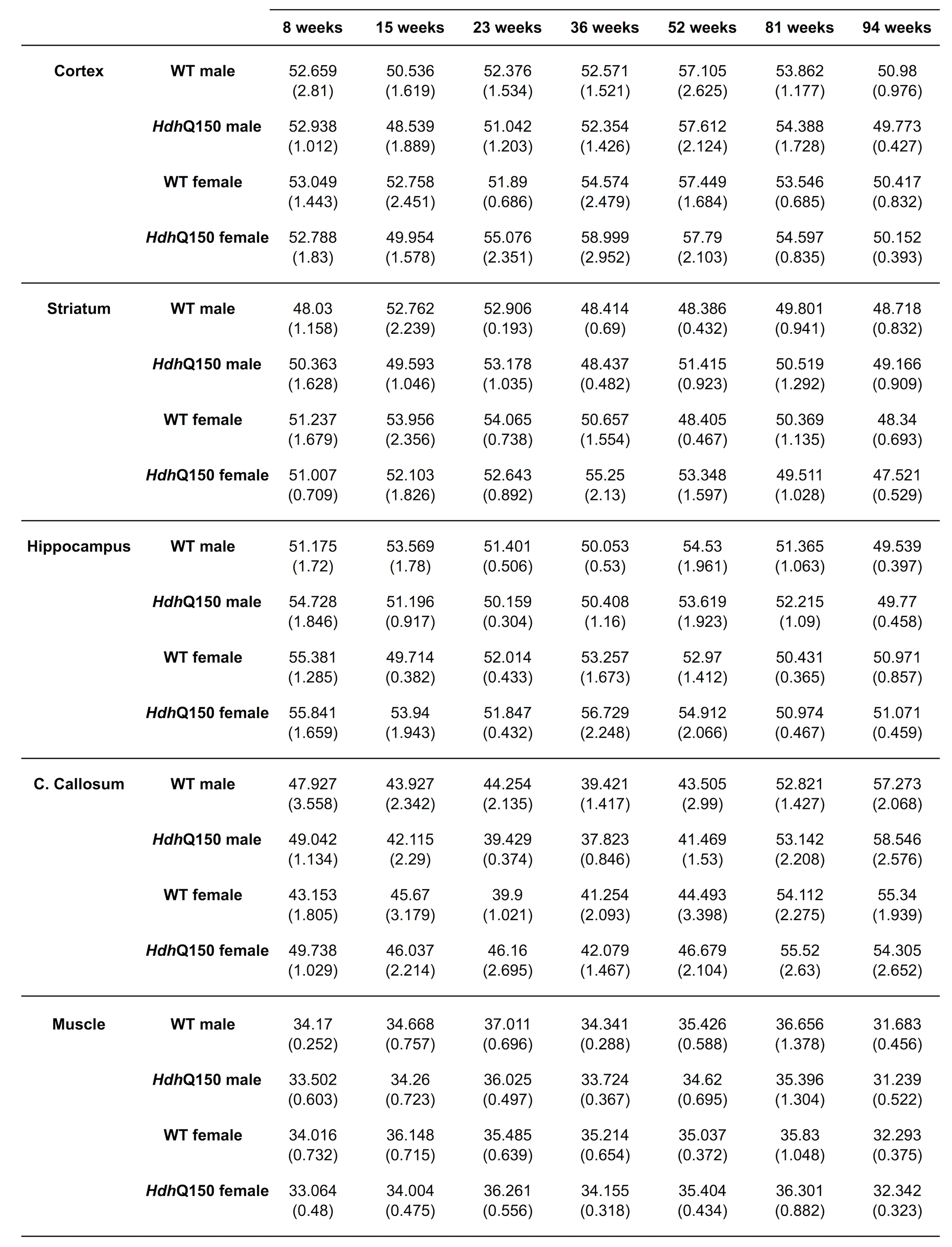

Supplement: S5 Table — Mean (SEM) T2 relaxation times (msec) for four brain regions and cheek muscle tissue across the seven in vivo MRI scans. (TIFF) [file pone.0168556.s005.tiff]

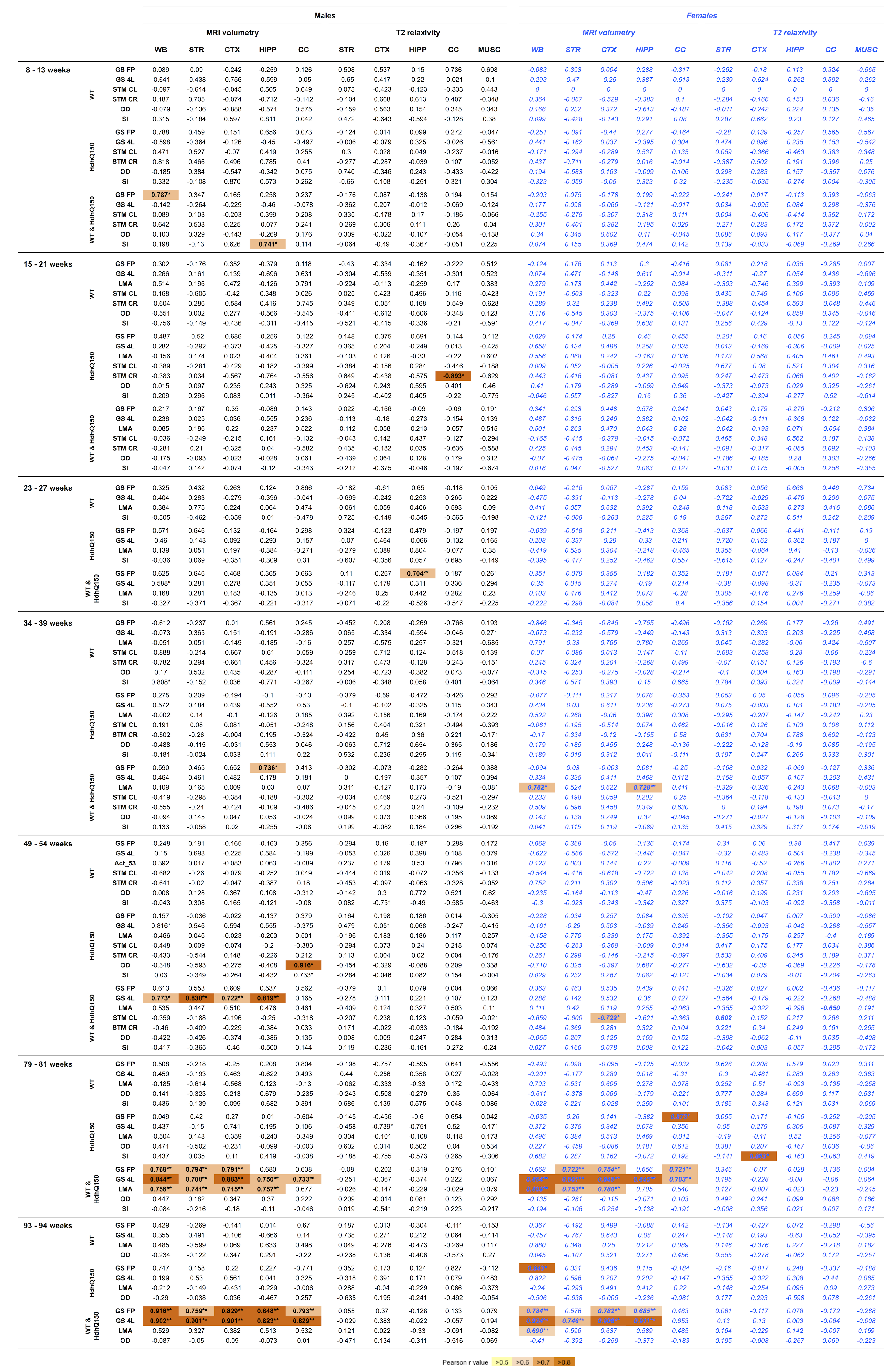

Supplement: S6 Table — Correlations of behavioral measures against age-matched MRI measures at six time points (8–13 weeks, 15–21 weeks, 23–27 weeks, 34–39 weeks, 49–54 weeks, 79–81 weeks, 93–94 weeks) presented as Pearson r values. GS FL = fore limb grip strength, GS 4L = fore and hind limb grip strength, LMA = locomotor activity in an open-field, TM CL = cued learning in a swimming T-maze, TM CR = cue reversal learning in a swimming T-maze, OD = odor descrimination, SI = social interaction, WB = whole brain, STR = striatum, CTX = cortex, HIPP = hippocampus, CC = corpus callosum, MUSC = cheek muscle. *Statistically significant after Bonferroni Correction (adjusted p value: 8–13 weeks p = 0.0017; 15–21 weeks p = 0.0014; 23–27 weeks p = 0.0025; 34–39 weeks p = 0.0014; 49–54 weeks p = 0.0014; 79–81 weeks p = 0.002; 93–94 weeks p = 0.0025). (TIFF) [file pone.0168556.s006.tiff]

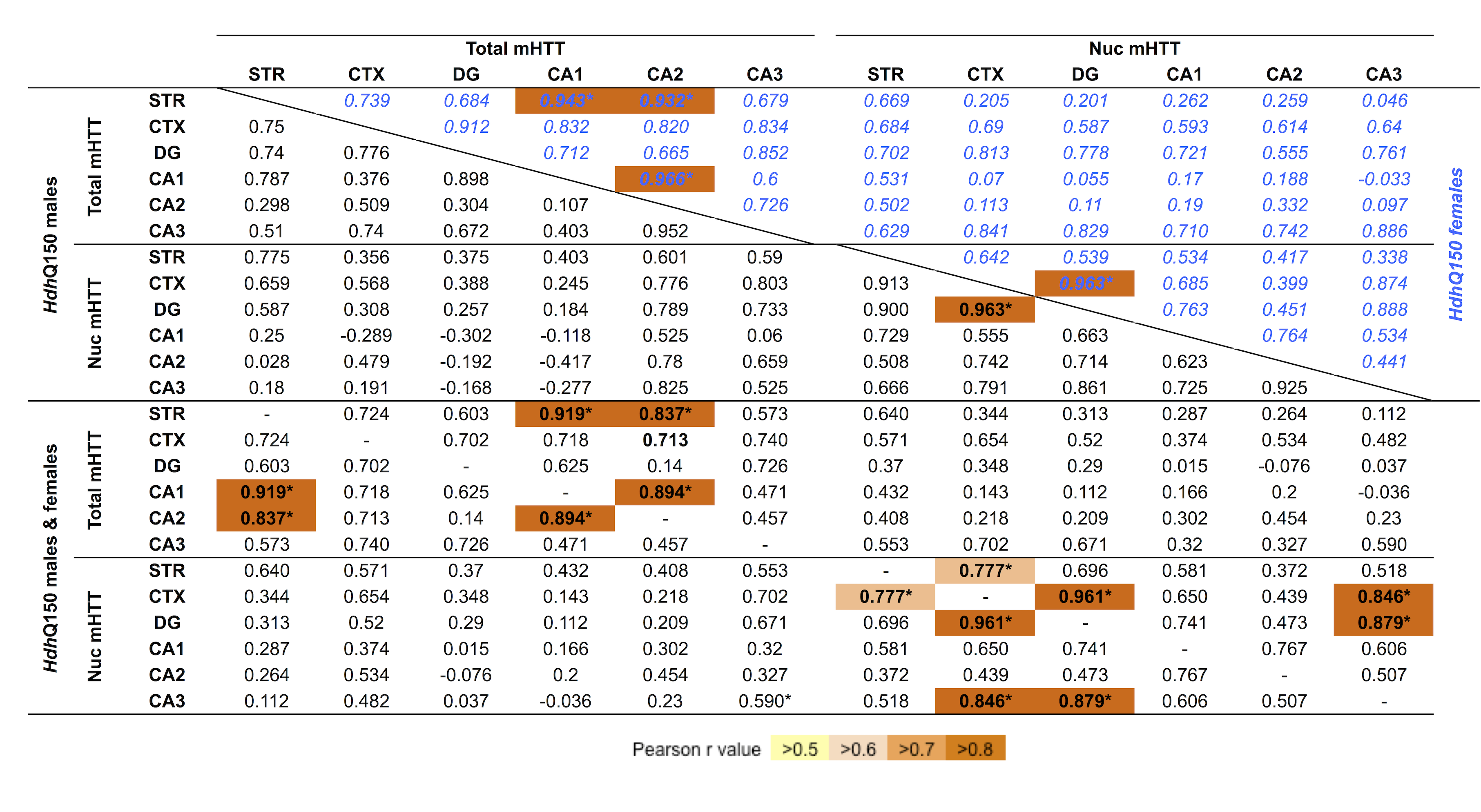

Supplement: S7 Table — Correlations of total aggregated mHTT (Total mHTT) and nuclear mHTT inclusions (Nuc mHTT) measured on S830-stained sections, presented as Pearson r values. STR = striatum, CTX = cortex, DG = hippocampal dentate gyrus, CA1 = hippocampal CA1 subfield, CA2 = hippocampal CA2 subfield, CA3 = hippocampal CA3 subfield. *Statistically significant after Bonferroni Correction (adjusted p value 0.0008). (TIFF) [file pone.0168556.s007.tiff]

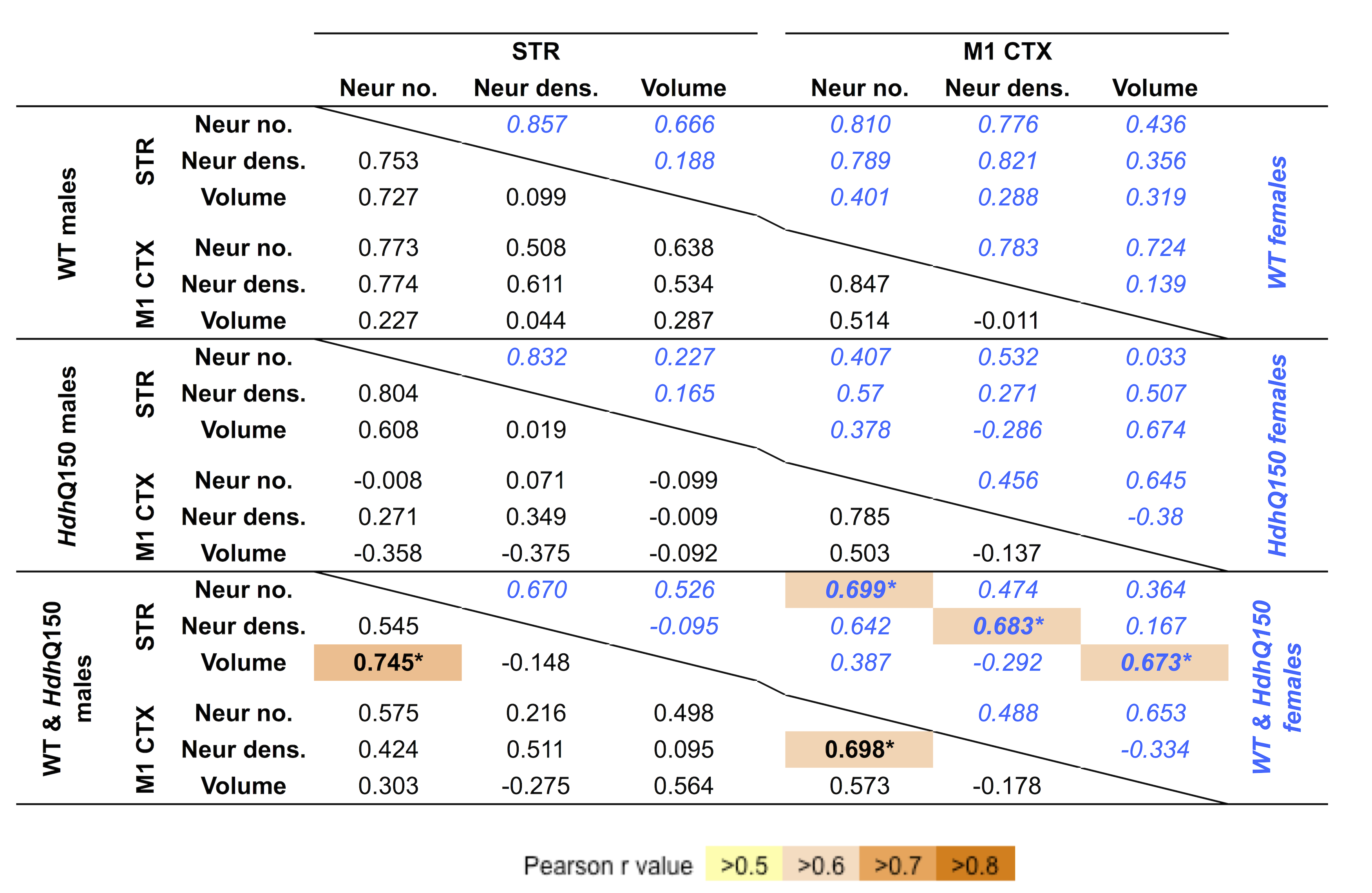

Supplement: S8 Table — Correlation of stereological measures of neuronal characteristics on NeuN-stained brain sections, presented as Pearson r values. STR = striatum, M1 CTX = M1 cortex, Neur no. = neuronal number, Neur dens. = neuronal density. *Statistically significant after Bonferroni Correction (adjusted p value 0.003). (TIFF) [file pone.0168556.s008.tiff]

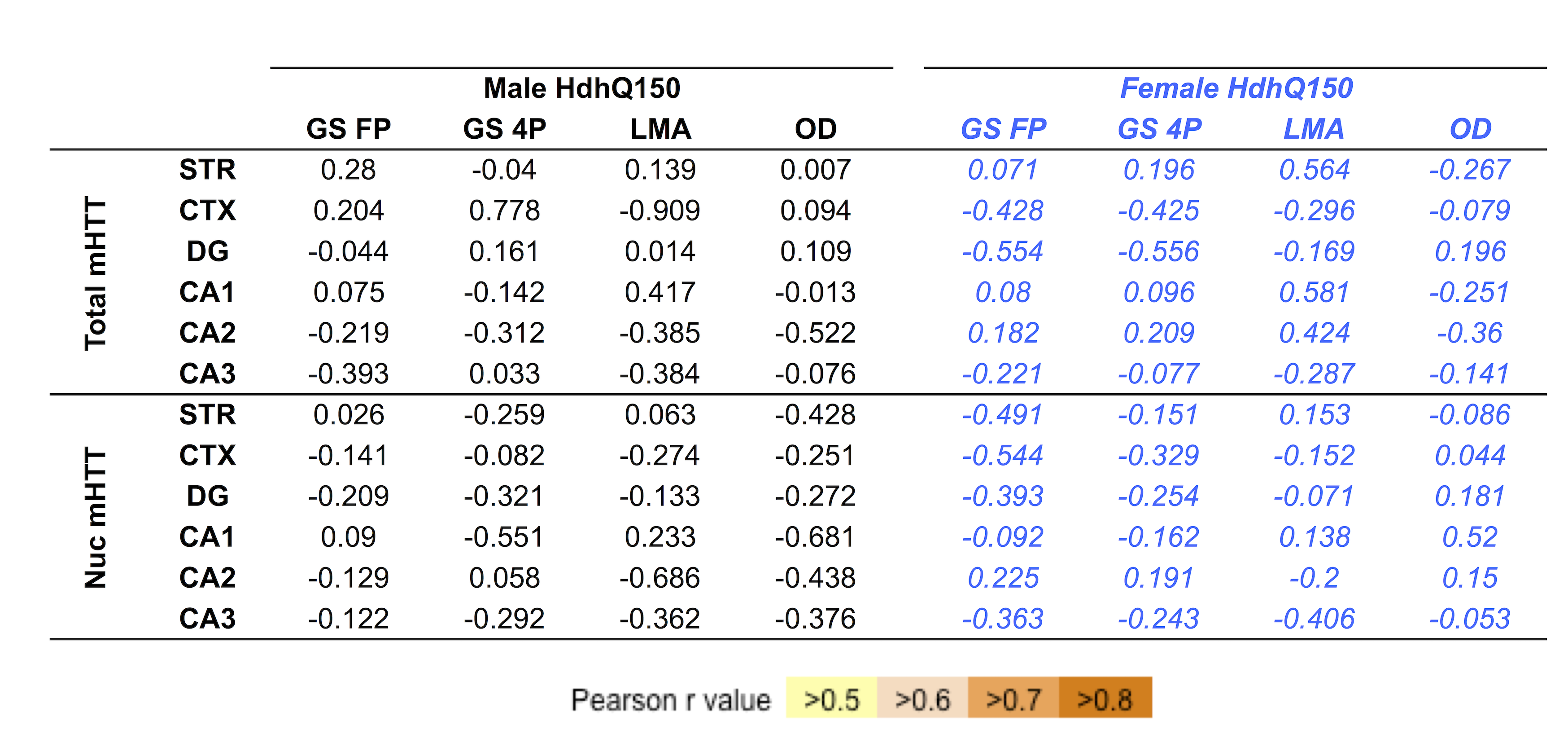

Supplement: S9 Table — Correlations of total aggregated mHTT (Total mHTT) and nuclear mHTT inclusions (Nuc mHTT) versus behavioral performance at the final time point (92–94 weeks). STR = striatum, CTX = cortex, DG = hippocampal dentate gyrus, CA1 = hippocampal CA1 subfield, CA2 = hippocampal CA2 subfield, CA3 = hippocampal CA3 subfield, GS FL = fore limb grip strength, GS 4L = fore and hind limb grip strength, LMA = locomotor activity in an open-field, OD = odor descrimination. There were no significant correlations after Bonferroni Correction (adjusted p volue 0.002). (TIFF) [file pone.0168556.s009.tiff]

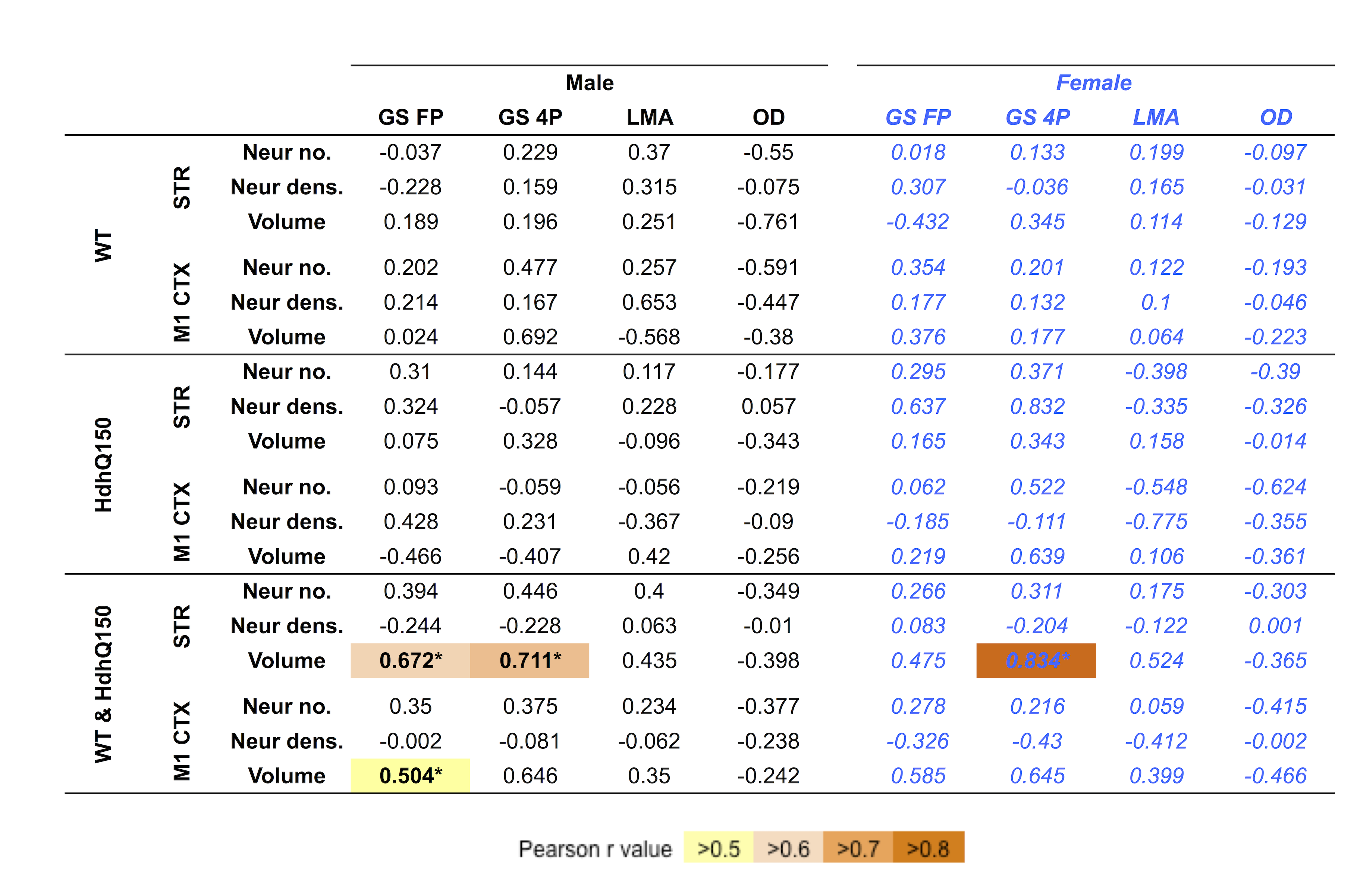

Supplement: S10 Table — Correlations of stereological measures of neuronal characteristics versus behavioral performance at the final time point (92–94 weeks). STR = striatum, M1 CTX = M1 cortex, Neur no. = neuronal number, Neur dens. = neuronal density, GS FL = fore limb grip strength, GS 4L = fore and hind limb grip strength, LMA = locomotor activity in an open-field, OD = odor descrimination. *Statistically significant after Bonferroni Correction (adjusted p value 0.0042). (TIFF) [file pone.0168556.s010.tiff]

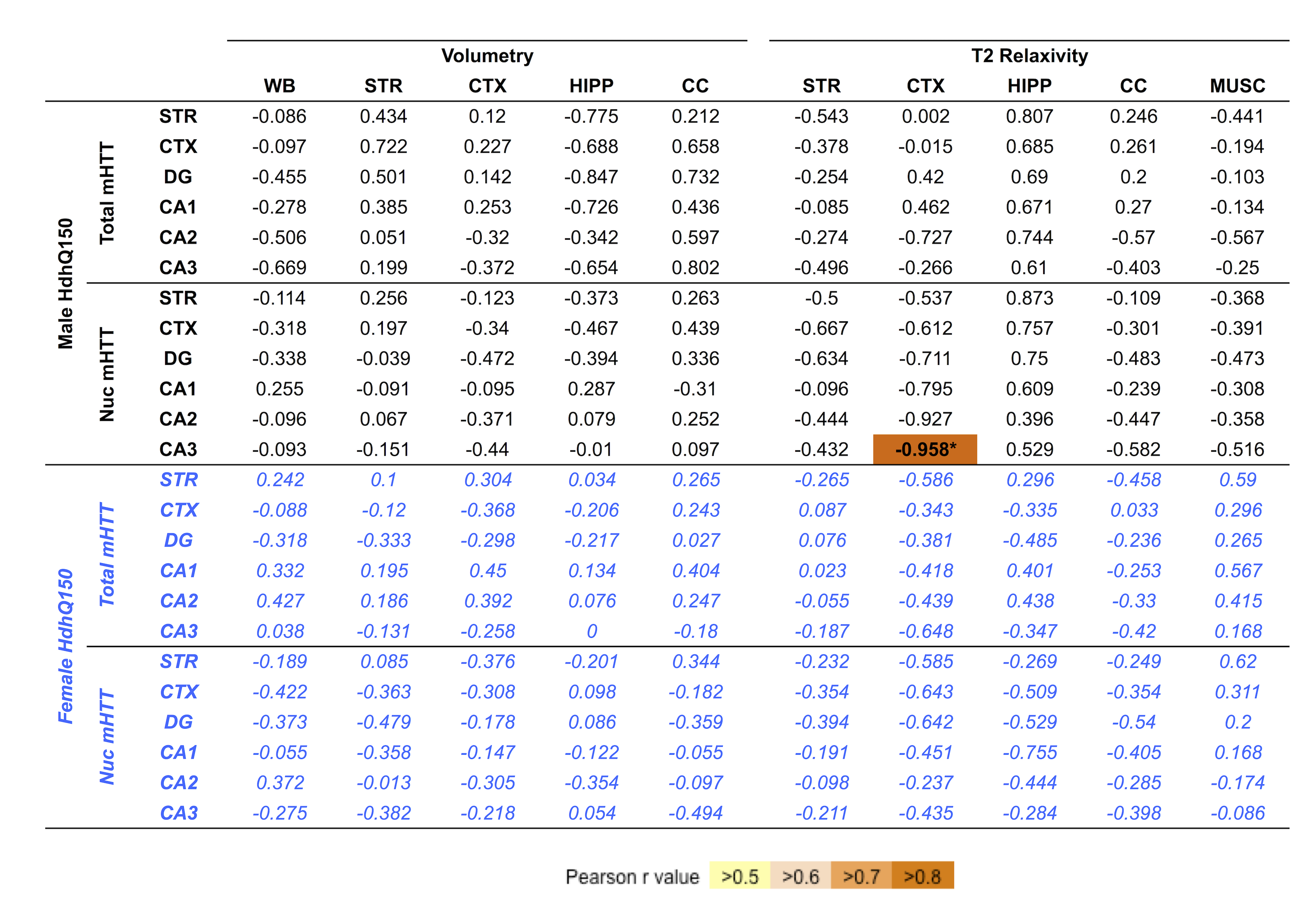

Supplement: S11 Table — Correlations of total aggregated mHTT (Total mHTT) and nuclear mHTT inclusions (Nuc mHTT) versus MRI measures taken at the final in vivo time point (94 weeks). STR = striatum, CTX = cortex, DG = hippocampal dentate gyrus, CA1 = hippocampal CA1 subfield, CA2 = hippocampal CA2 subfield, CA3 = hippocampal CA3 subfield, WB = whole brain, HIPP = hippocampus, CC = corpus callosum, MUSC = cheek muscle. *Statistically significant after Bonferroni Correction (adjusted p value 0.0017). (TIFF) [file pone.0168556.s011.tiff]

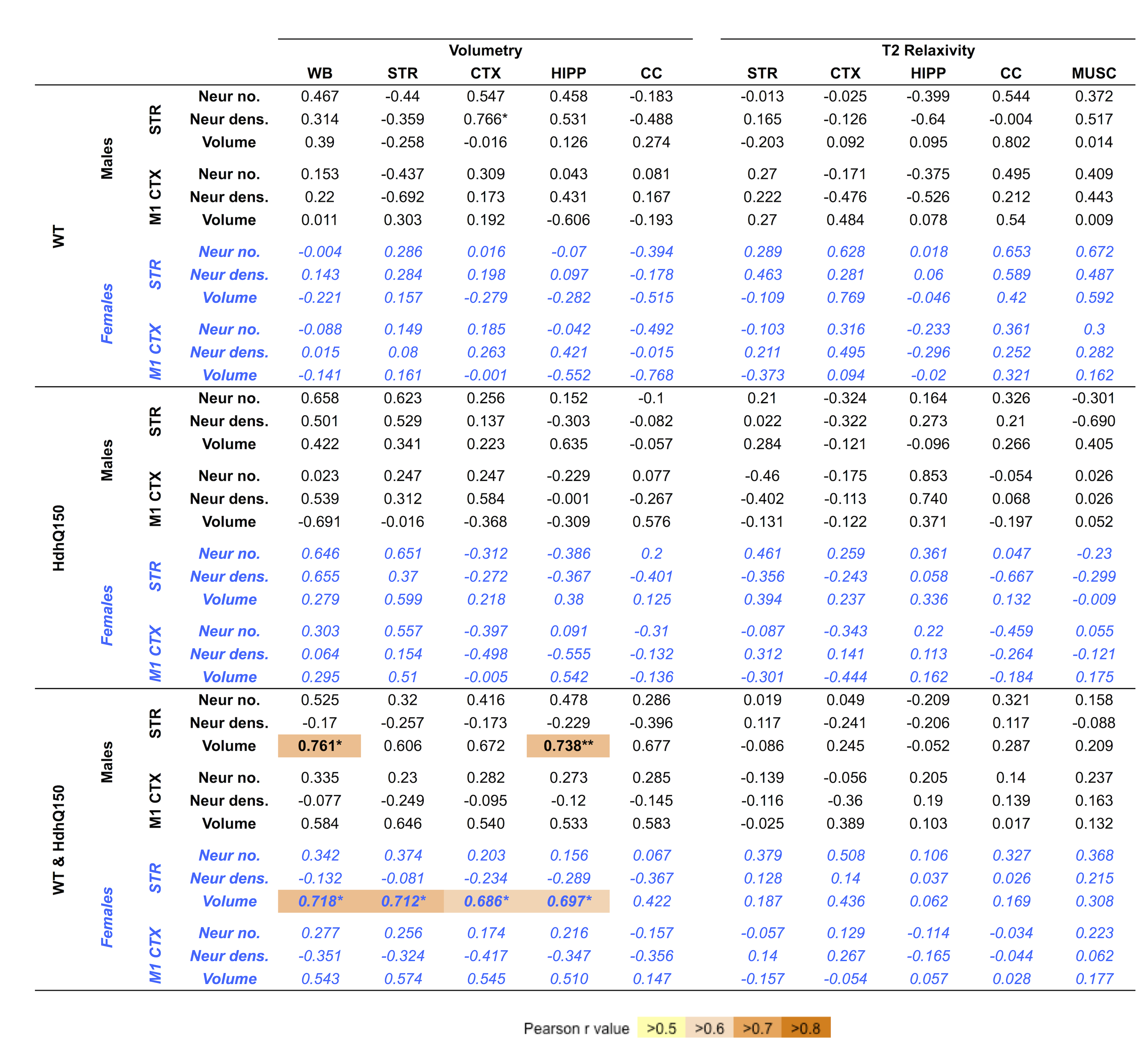

Supplement: S12 Table — Correlations of stereological measures of neuronal characteristics versus MRI measure of pathology taken at the final in vivo time point (94 weeks), presented as Pearson r values. STR = striatum, M1 CTX = M1 cortex, Neur no. = neuronal number, Neur dens. = neuronal density, WB = whole brain, CTX = cortex, HIPP = hippocampus, CC = corpus callosum, MUSC = cheek muscle. *Statistically significant after Bonferroni Correction (adjusted p value 0.003). (TIFF) [file pone.0168556.s012.tiff]
